# The *Surales*, Self-Organized Earth-Mound Landscapes Made by Earthworms in a Seasonal Tropical Wetland

**DOI:** 10.1371/journal.pone.0154269

**Published:** 2016-05-11

**Authors:** Anne Zangerlé, Delphine Renard, José Iriarte, Luz Elena Suarez Jimenez, Kisay Lorena Adame Montoya, Jérôme Juilleret, Doyle McKey

**Affiliations:** 1 Technische Universität Braunschweig (TUB), Institut für Geoökologie, Langer Kamp 19c, D-38106, Braunschweig, Germany; 2 CEFE UMR 5175, CNRS – Université de Montpellier – Université Paul Valéry Montpellier – EPHE, 1919 route de Mende, 34293 Montpellier Cedex 5, France; 3 Department of Geography and Natural Resource Sciences, McGill University, Montreal, QC, H3A 2T5, Canada; 4 Department of Archaeology, College of Humanities, University of Exeter, Exeter, EX4 4QE, United Kingdom; 5 Fundacion Universitaria Internacional del Tropico Americano (Unitropico), Cra. 19, Yopal, Colombia; 6 Laboratorio de Perifíton, CEP: 87020–900 – Maringá – PR Universidade Estadual de Maringá (UEM), Av. Colombo, 5.790 – Bloco G-90, Sala 8, Maringá, Brazil; 7 Luxembourg Institute of Science and Technology, Environmental Research and Innovation Department, 41 rue du Brill, L-4422, Belvaux, Luxembourg; 8 Institut Universitaire de France, Paris, France; University of Roehampton, UNITED KINGDOM

## Abstract

The formation, functioning and emergent properties of patterned landscapes have recently drawn increased attention, notably in semi-arid ecosystems. We describe and analyze a set of similarly spectacular landforms in seasonal tropical wetlands. *Surales* landscapes, comprised of densely packed, regularly spaced mounds, cover large areas of the Orinoco Llanos. Although descriptions of *surales* date back to the 1940’s, their ecology is virtually unknown. From data on soil physical and chemical properties, soil macrofauna, vegetation and aerial imagery, we provide evidence of the spatial extent of *surales* and how they form and develop. Mounds are largely comprised of earthworm casts. Recognizable, recently produced casts account for up to one-half of total soil mass. Locally, mounds are relatively constant in size, but vary greatly across sites in diameter (0.5–5 m) and height (from 0.3 m to over 2 m). This variation appears to reflect a chronosequence of *surales* formation and growth. Mound shape (round to labyrinth) varies across elevational gradients. Mounds are initiated when large earthworms feed in shallowly flooded soils, depositing casts that form ‘towers’ above water level. Using permanent galleries, each earthworm returns repeatedly to the same spot to deposit casts and to respire. Over time, the tower becomes a mound. Because each earthworm has a restricted foraging radius, there is net movement of soil to the mound from the surrounding area. As the mound grows, its basin thus becomes deeper, making initiation of a new mound nearby more difficult. When mounds already initiated are situated close together, the basin between them is filled and mounds coalesce to form larger composite mounds. Over time, this process produces mounds up to 5 m in diameter and 2 m tall. Our results suggest that one earthworm species drives self-organizing processes that produce keystone structures determining ecosystem functioning and development.

## Introduction

The increasing ability to screen the Earth‘s surface from the air reveals how common and diverse regular patterns are in nature. Regularly spaced termite mounds and vegetation patterns in semi-arid environments have prompted most of the research aimed at understanding the mechanisms that cause patterning in landscapes and their emergent ecological properties (e.g. [1–4]). Although patterned landscapes featuring earth mounds are widespread in seasonal tropical wetlands of South America [5], they have attracted much less attention from ecologists. Among the least known are “*surales*” landscapes, which must be seen to be believed. They are found in the Orinoco Llanos of Colombia and Venezuela, mostly in the seasonally shallowly flooded waters of the alluvial overflow plain [6]. Bates ([7] pages 566 and 568) provides a vivid description of these landscapes: “*The surales present a reticulate pattern of deep ditches surrounding mounds a meter or two in diameter; the top of the mound is a meter or more above the bottom of the surrounding ditch*. *… The reticulate ditching is like the pattern formed by the drying of a gigantic mud flat…Well-developed sural country is difficult to traverse; if you are on foot*, *you have to decide whether to follow the endless twistings of the boggy ditches or to jump from mound to mound*, *both awkward expedients*. *If you are mounted*, *the animal has to make the same decision and generally ends up in complete frustration*: *I have heard stories of man and mule firmly stuck in a narrow*, *deep ditch between two sural mounds*.”

Although *surales* are mentioned in numerous studies of plant ecology in the Llanos, only few published studies focus specifically on them (see for example [8]). Given the importance of topographic heterogeneity in the functioning of wetland ecosystems (e.g. [9,10]), it is astonishing that no work, even at the most basic levels, has been done to examine the ecological consequences of the pronounced micro-relief of *surales* landscapes. In this article, we provide the first complete description of *surales*. Using satellite imagery and results from fieldwork, we provide insights into the spatial extent of *surales* landscapes and the mechanisms that are responsible for the formation and growth of *surales* mounds. In addition, we observed a continuum of landscapes, in which mounds show great variation across sites in form (from round mounds to labyrinths) and size. Here, we explore this variation as a result of environmental variation and as a process of development over time that leads to increasing topographic heterogeneity, which in turn affects the distribution of plants and other organisms in *surales* landscapes.

Patterned landscapes in a great range of environments, from estuarine marshes to boreal peatlands and arid and semi-arid shrublands, are studied using the concept of spatial self-organization in ecosystems [3,11]. This concept links scale-dependent feedbacks (positive at small scale and negative at larger scale) between organisms and environment to the emergence across entire landscapes of regular patchiness of resources and of organisms that depend on them [12]. Recent research on patterned landscapes in semi-arid ecosystems has highlighted the role of organisms considered to be soil “ecosystem engineers” [13], such as social insects and plants, in driving spatial self-organization [1,2]. Variation in the scale, strength and orientation of feedbacks driven by these ecosystem engineers explains the diversity of the shape (i.e., round spots, parallel stripes and labyrinths) and size of the emergent patterns visible in vegetation [3,14,15].

In seasonal tropical wetlands, patterned landscapes show similar repeated motifs of earth mounds that vary considerably in size and spacing among different landscapes [5]. Despite apparent parallels with patterned ecosystems elsewhere, these earth-mound landscapes have not yet been studied within the conceptual framework of self-organization, even though the formation of mounds often involves the driving role of soil ecosystem engineers. Although purely physical mechanisms (swelling and shrinking of smectite clays in vertisols leading to development of gilgai microtopography [16,17]) may explain the formation of mounds in a few particular environments, most hypotheses envisage a driving role of organisms. Two different hypotheses ascribe to organisms very different roles. The first hypothesis posits the importance of animals as mound builders. Although there is good evidence for mound construction in seasonal tropical wetlands by social insects (e.g., termites in Australia [18], Africa and South America [19]; *tacurú* landscapes built by *Camponotus punctulatus* ants in Argentina [20]) and, in seasonally waterlogged temperate prairies, by burrowing mammals [21], the building-up hypothesis has been challenged [22–25]. Alternative hypotheses have been proposed, notably in attempts to explain the formation of *murundu* mounds in seasonally waterlogged cerrado in central Brazil. Furley [26] and Silva et al. [25] posit that mounds result from the differential erosion of an initially higher land surface, in which ‘mounds’ are formed where roots of regularly spaced woody plants stabilize the soil against erosion (cf. [22,23] for other landscapes). In this alternative hypothesis, the key organisms are not mound-building animals, but rather the plants. Why the plants are regularly spaced has not been addressed in these cases.

Because descriptions of *surales* mounds suggest that they consist largely of the accumulated excreta of earthworms, most studies ascribe their origin, rather matter-of-factly, to the activities of these soil ecosystem engineers: “In the Neotropical savannas, on the humid savannas (hyperseasonal savanna), the activities of the earthworms responds [*sic*] not only to the soil moisture but also to the flooded conditions, and during the flood the earthworm produces an earth mound in order to search for aeration” [27]. The terms *tatuco*, *zuro*, *zural* and *sural* are all used interchangeably in different parts of the Orinoco Llanos to refer to mounds literally covered by earthworm casts, or to landscapes featuring these mounds [28–31]. In Venezuela, *surales* are often known as *lombrizales*, a name alluding to their likely origin resulting from the activities of earthworms. However, although the role of earthworms in generating *surales* topography is widely accepted, (i) the identity of the earthworm(s) thought to produce these landscapes has never been examined and (ii) in some environments earthworms and termites are both abundant, and distinguishing their constructions is not always straightforward. As for several other kinds of mound-field landscapes, the alternative hypothesis that mounds result from differential erosion has also been proposed for *surales*. Goosen [32] ascribed the origin of *surales* landscapes to “reticular gully erosion” (see for example sections beginning p. 42, p. 130). However, in our opinion he fails to explain in a cogent manner how such a process could work, and how it could result in the highly regular distribution of mounds. In the areas where *surales* landscapes have been found, slope is very slight (less than 1%); the savanna is flooded by rainwater, and only exceptionally by flowing river water; there is no active sedimentation and, even in the higher parts of the Llanos, no severe dissection of terrain [32]. In addition, we found no authoritative source that demonstrates this phenomenon or even offers an explanation of how it could work. Finally, those who follow Goosen [32] in positing a contribution of “reticular erosion” to the formation of *surales* landscapes still ascribe an important role to earthworms [29], as did Goosen [32] himself, somewhat hesitantly, in the penultimate chapter of his thesis (p. 137).

In this paper, we address the following questions: (1) What is the geographical distribution of *surales* landscapes in the Orinoco Llanos of Colombia and Venezuela? (2) What are the mechanisms that form *surales*, and what roles are played by soil ecosystem engineers? (3) What processes lead to the diversity of size and form of *surales* mounds observed across landscapes? (4) How does the topographic heterogeneity of *surales* landscapes affect communities of plants and earthworms? To answer these questions, we combined remote sensing techniques (based on satellite imagery available in Google Earth^™^, and aerial photography using a drone) with field studies comparing a range of sites. We measured soil physical and chemical properties and studied soil macrofauna and the effects of their activities on bioturbation and on soil structure. We analyzed plant microfossils in soils to attempt to gain insight into vegetation history, and surveyed plant biodiversity within and among sites. Based on our findings, we postulate a mechanism by which feedbacks resulting from activities of earthworms lead to spatial regularity of *surales* mounds and development of *surales* landscapes over time.

## Materials and Methods

### The geological and geomorphological setting

Large inland wetlands occupy over one million square kilometers in South America [33]. The largest of these are found in subsidence basins to the east of the Andes—the Orinoco Llanos, the Bolivian Llanos and the Pantanal—where Andean uplift led to erosion, sedimentation and altered climates, creating some of the world’s largest flooded grasslands [6,33–35]. Hamilton et al. [34] found that among years (over an 8-year period) seasonal flooding affected from around 60,000 km^2^ to 105,000 km^2^ of the Orinoco Llanos (mean around 80,000 km^2^). Within the Orinoco Llanos, *surales* landscapes are found in the physiographic province called the ‘alluvial overflow plain’. This region is a flat plain (elevation about 180 m asl in our study sites) in which slight differences in elevation lead to large differences in flooding regimes. Low-lying areas near streams, which account for 5–10% of the alluvial overflow plain [32], include permanent ponds (*esteros*) covered by water much of the year. Natural levees and splays (60% of the alluvial overflow plain) include sandy *bancos*, which are never flooded. Located in ‘slackwater areas’ [32] between these two extremes, hyperseasonal savannas are characterized by both dry-season drought and shallow flooding in the rainy season [27]. *Surales* landscapes are particularly frequent in these environments. Although soils in the alluvial overflow plain show some variation [6], they all are characterized by the dominance of kaolinitic clays, low cation exchange capacity and strong hydromorphism, with the appearance of perched water tables near the surface during the rainy season [36].

### Estimation of the spatial extent of *surales* landscapes

We estimated the spatial extent of *surales* landscapes in the area covered by the Orinoco Llanos by combining various methods: (i) we collected information on sites georeferenced in the soil science and ecology literature; (ii) we added the information from our own field sites and from sites whose locations were communicated to us by other researchers working in this region; (iii) based on our expertise of this landform from the ground, we conducted a systematic survey of Google Earth^™^ satellite images. To do this, we defined a grid at 50 x 50 km intervals within the boundaries of the Orinoco Llanos (as defined in the World Wildlife Fund’s Encyclopedia of Earth [http://www.eoearth.org/view/article/154273/]) using the open source software QGIS 2.6.1-Brighton [37]. Each node of the grid represented an observation site (n = 125) around which a circle of 5 km radius was defined. The area within each of these circles (78.54 km^2^) was exhaustively surveyed for the presence (score of 1) or absence (score of 0) of *surales* landforms. Using this methodology, we surveyed 9,817 km^2^ of the 376,547 km^2^ covered by the Orinoco Llanos. We interpolated the binary data obtained on the regular grid to estimate the probability of *surales* presence at the scale of the Orinoco Llanos. To do this, we used a thin plate spline regression method, suited for data that do not respect the hypothesis of stationarity required for statistical methods such as kriging. Our analysis did not take into account surveyed points scored “NA”, i.e., points for which satellite imagery of sufficient resolution was not available. To calculate the estimated area of the Orinoco Llanos that features patches of *surales*, we counted the number of pixels (pixel size: 443.67 x 443.67 m) of the Orinoco Llanos that were ascribed an interpolated value > 0.5. In order to show a sample of some of the most spectacular sites discovered doing the satellite imagery survey, we created a commented tour using Google Earth Pro^™^ and its movie maker module https://mycore.core-cloud.net/public.php?service=files&t=e01a65c5769726d35c4f005a13c46502.

### Field sites and sampling design

Our study was conducted in the seasonally flooded savannas of the municipality of San Luis de Palenque, Casanare, Colombia, which is included in the alluvial overflow plain. Field studies were carried out on privately owned land with the permission of landowners, and did not involve endangered or protected species. Climate is seasonal humid tropical; mean annual temperature is 28°C, mean annual rainfall is 2000 mm. An intense rainy season occurs from May to July, followed in most years by a short dry period, of varying duration and intensity, in August and September. A less intense rainy season occurs from October to mid-November, followed by an intense dry season from December to April.

**Fig 1:**
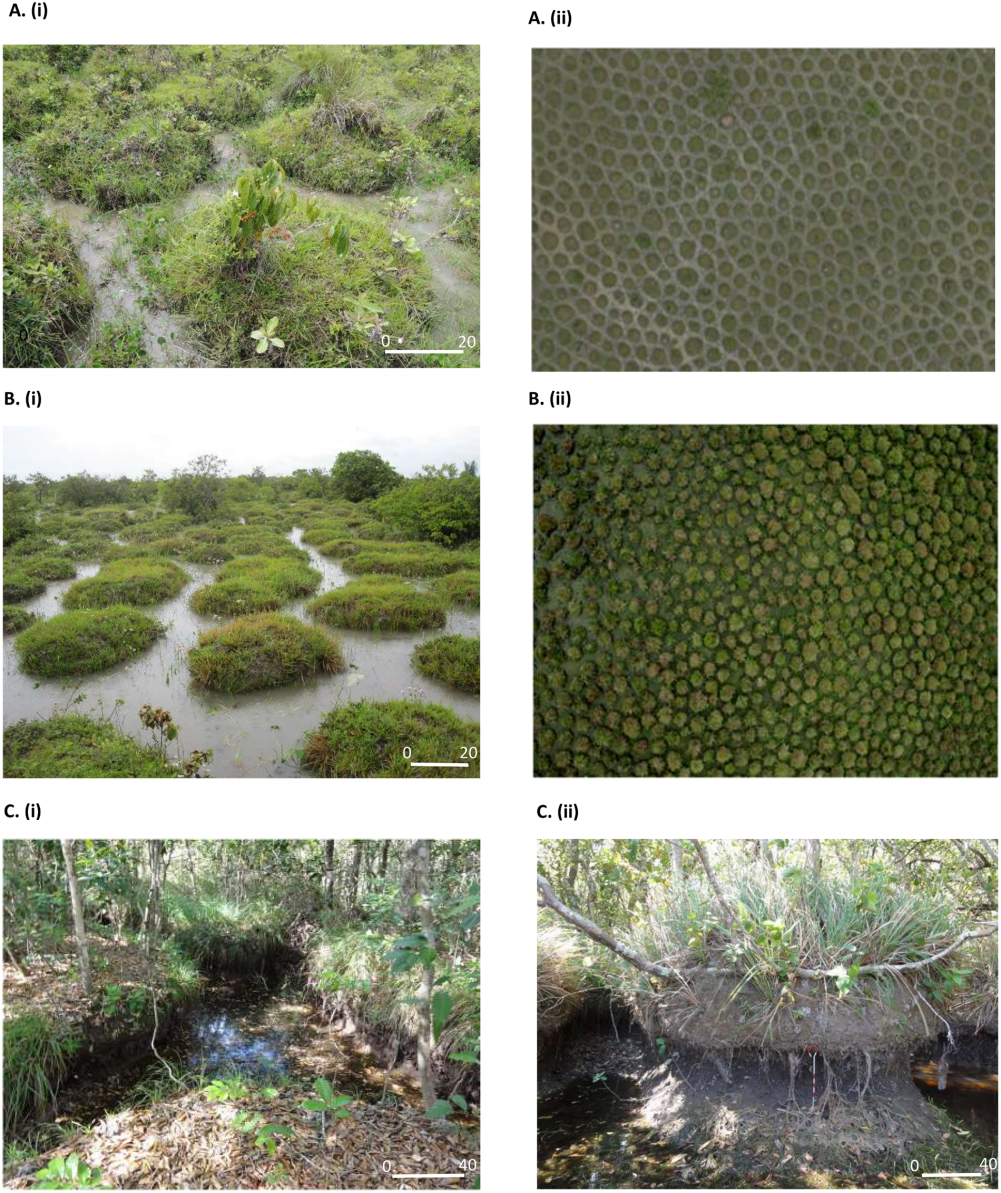
Aerial and terrestrial images of the three field sites bearing *surales*. A. Site 1, B. Site 2, C. Site 3. Aerial images were taken using the Pixy^™^ drone, Delphine Renard 2012. A(i), B(i): Photo Doyle McKey 2012; C(i), C(ii): Photo José Iriarte 2012.

During a first field mission in July 2012, we distinguished three main categories of round- mound *surales* landscapes, characterized by differences in mound sizes, in the depth of the inter-mound basin, in duration of the flooding period and in vegetation cover (Fig 1). Consequently, we investigated the ecology of *surales* in three field sites, spanning the different categories of *surales* landscapes. Site 1 (N 05°17”12.3’; W 71°57”44.8’) was characterized by the smallest mounds, with an average (± SD) height of 37.5 ± 7.3 cm (distance from the base of the inter-mound basin to the top of the mound) and an average diameter of 112 ± 47.1 cm. In Site 2 (N 05°13”22.6’; W 71°58”11.5’) the size and shape of *surales* mounds varied among locations within the site. In the area within this site that we studied, mounds averaged 61.7 ± 10.8 cm in height and 160.6 ± 32.5 cm in diameter. Site 3 (N 04°55”37.4’; W 71°59”10.9’), in contrast to the others, was covered by trees and mounds were very large. In the part of this site we studied, the average height of mounds was 114.7 ± 17.6 cm and the average diameter was 373 ± 109 cm. Elsewhere in the site, some mounds reached 2 m in height and up to 5 m in breadth. Site 4 (N 04°55”32.5’; W 71°59”09.3’) was considered a control or reference site. It was located close to Site 2 on a *banco*, i.e., a bank on higher ground that is not seasonally flooded and where *surales* mounds are absent. The sites investigated are all semi-natural grasslands, mainly used for extensive cattle grazing.

In each of Sites 1–4, we collected samples to obtain data on soil properties, earthworm species composition and abundance and plant microfossils (phytoliths) in soil, in three plots of 25 m^2^, each separated from the others by a distance of 30 meters representing an equilateral triangle. No large termite nests were present in any of the sites we studied. Samples for soil properties and earthworm species composition and abundance were collected in five sampling locations within each plot. In *surales* habitat (Sites 1–3), three of the five sampling locations in each plot were located on mounds and two in the inter-mound basin, giving a total of 27 sampling locations for mounds (nine in each site) and 18 sampling locations for inter-mounds (six per site). Sampling locations in each plot were separated by varying distances, as a function of differences between sites in size and spacing of mounds. In Site 4, three plots (and 15 sampling locations) were located in apparently homogeneous *banco* habitat. We surveyed earthworm abundances and spatial distribution in mound and inter-mound habitats during two field missions at the end of the rainy season, in July 2012 and July 2013. At these times, soils were still wet but the inter-mound basin was no longer completely flooded. In these conditions, earthworms were active and close to the soil surface in the mounds, facilitating sampling. A sampling campaign was also conducted during the dry season in December 2012 to study phytolith composition, soil properties and morphology of soil macroaggregates in the different study sites. At this period the inter-mound basin was not flooded, except for Site 3, in which the basin is flooded all year round.

### Characterization and analysis of plant communities

Plant communities were sampled during the dry season in December 2012 and again, using the same sites and methods, during the rainy season in July 2013, when the inter-mound basin was flooded. Plant species composition and distribution were recorded on *surales* mounds and in inter-mound basins for Sites 1 to 3, as well as in a control (reference) site in a *banco*. For logistical reasons, *banco* vegetation (never flooded) was sampled only during the rainy season (July 2013) and not in Site 4, but in a nearby site, Site 5 (N 04°55”36.8’; E 71°59”12.5’), a natural levee located close to Sites 2 and 4. Vegetation was sampled using the point-intercept method [38], which provides an estimation of the contribution of each species to vegetation cover (i.e., cover abundance). In Sites 1, 2 and 5, we sampled two parallel 20-m-long line transects, separated by a distance of 10 m. Only one transect was sampled in Site 3. Every 20 cm, we recorded and identified each plant contacted by a line extending vertically from the transect line. Also, for each of the 700 sampling points, a score of 1 was given when we contacted at least one plant and a score of 0 was given when the vertical projection encountered only bare soil. In the sites bearing *surales* (Sites 1–3), habitat type (mound or inter-mound) was also recorded for each sampling point. In Site 5, the *banco* habitat was apparently homogeneous.

Whereas vegetation in Sites 1, 2 and 5 was strongly dominated by graminoids, along with some herbaceous and small woody dicots, Site 3 was dominated by trees averaging about five meters in height. In this site, in addition to the point-intercept method, all trees with a diameter at breast height (DBH) greater than 10 cm were sampled using a line transect method [39] along a 50-m transect extending one meter each side of the line, i.e., in an area of 100 m^2^ comprising both mound and inter-mound habitats.

We collected voucher specimens of each plant species encountered and identified plants using published keys [40–53] and by comparison with specimens in the online collections of the National Herbarium of Colombia (http://www.biovirtual.unal.edu.co/ICN/) and of the *Herbario Forestal Gilberto Emilio Mahecha Vega* (Universidad Distrital Francisco José de Caldas, Bogotá, http://herbario.udistrital.edu.co/herbario/). Sterile specimens that could not be identified were assigned to morphospecies.

We performed statistical analyses of plant community structure using only species that accounted for 5% or more of the vegetation cover (as estimated by the line-intercept method) in at least one of the three habitats (mounds, inter-mound basins, *banco*), in at least one of the four sites. For analysis, we grouped all successive points lying within the same habitat type into a single unit (referred to as a ‘segment’). To do this, we summed the number of times each plant species was contacted on each mound and inter-mound segment and divided the sum by the total number of contacts recorded in the segments of each type in the site. We analyzed a total of 42 mound and 37 inter-mound segments for the dry season and 44 mound and 41 inter-mound segments for the rainy season. This method allowed controlling for the difference in sampling efforts due to the differing representation of mound and inter-mound areas in the landscape.

We used a non-metric multidimensional distance scaling analysis (NMDS) based on a Bray-Curtis dissimilarity matrix to assess differences in species composition and assemblage structure between habitats (mounds and inter-mounds) and among sites (Sites 1, 2 and 5). We performed separate NMDS analyses for the dry and the rainy seasons. Site 5 was included only in the analysis of the rainy season. Site 3 was not included in the analysis because it shared no plant species (in either habitat) with the other sites in the dry season and only two species, both at low abundance in Site 3, in the rainy season. To examine the contributions of site and of habitat type (nested factors) in explaining variation in the vegetation composition, we used a non-parametric multivariate analysis of variance (MANOVA) based on the distance matrix we performed for the NMDS. This test was followed by a permutation test (*n* = 999 permutations). To study the distribution of different plant life forms between mounds and inter-mounds and among sites, plant species records were grouped as herbaceous plants, including “graminoids” (Poaceae, Cyperaceae, Eriocaulaceae), “aquatic herbs” and “other herbaceous species” (including one herbaceous climber); as suffrutescent plants (herbaceous, but with woody basal stems); and as woody plants, including “trees” and “other woody plants”, the latter consisting of shrubs as well as three woody vines. Cover abundance was scored for each species and grouped for each life form. All the statistical analyses conducted during this study were performed using the software R version 3.1.1 [54].

### Phytoliths

Phytoliths offer important advantages for studying vegetation histories in soil-depth profiles because (i) being made of silica, they are preserved in the hot-humid conditions and acid soils of the tropics where other types of plant remains are not [55] and (ii) they are deposited from plant materials that decay in place, and thus give a picture of vegetation composition at a highly local scale [56,57]. In order to investigate the vegetation history of the *surales* sites selected, we carried out phytolith analysis on a total of 58 samples collected from soil-depth profiles dug in the three sites bearing *surales*. In addition, we analyzed four samples, each composed of a single earthworm cast. In each of the sites, we selected representative mound and inter-mound sampling points along the trenches cut for the study of soil profiles (see section 6 above). In each trench we took samples from profiles at two points: (i) at the highest central point of the mound and (ii) in the lowest central point of the basin. At Sites 1 and 2, we took samples at 5 cm intervals from the surface to 40 cm b.s. (below surface) (see S1 Fig). At Site 3, we took samples at 5 cm intervals down to 30 cm b.s and then at 10 cm intervals down to 110 cm b.s. Waterlogged conditions made it impossible to collect samples from deeper in the profile. Samples from the flooded basin were collected with a Russian corer. We collected earthworm casts for phytolith analysis from the soil surface on four haphazardly selected mounds of Site 1.

Soil samples were processed, and phytoliths identified and counted, at the University of Exeter Archaeobotany Laboratory following standard procedures [57]. A minimum of 200 phytoliths were counted per slide. Phytoliths were identified and counted under a Zeiss Axioscope 40 light microscope at 500X magnification. Phytolith morphotypes were identified by comparison with our phytolith reference collection from tropical and subtropical South America [58,59]. When possible, we followed the criteria of the ICPN group for naming phytolith types [60]. Identification of phytolith morphotypes typical of different taxa within Poaceae (Panicoideae, Chloridoideae and *Aristida* sp.) was based on a morphological classification first proposed by Twiss et al. [61], and later modified or refined by various researchers by taking into account criteria based on three-dimensional morphology and other micromorphological features (e.g., [58,62–65]). Identification of phytolith morphotypes of the families Cyperaceae, Marantaceae and Asteraceae, as well as *Heliconia* sp. and *Trichomanes* sp., followed Piperno [57]. The summary category “arboreal phytoliths” includes globular granulate, faceted elongate and sclereid phytolith morphotypes [57]. Globular psilates and silicified anticlinal/polyhedral epidermal cells were counted, but results for these types are not presented due to their low taxonomic resolution. Phytolith diagrams were made using C2 software [66].

### Sampling of earthworms

Earthworms were sampled and counted in July 2012 (at Sites 1, 2 and 4) and July 2013 (at Site 3) using the standard Tropical Soil Biology and Fertility (TSBF) method ([67] *ISO* 23611–5:2011 standard) applied to soil monoliths (25 x 25 x 30 cm depth) collected from the 60 sampling locations described in section 3 (i.e., nine mound and six inter-mound locations in each of Sites 1–3, and 15 locations from *banco* habitat in Site 4). Earthworms were extracted from the soil blocks by hand sorting. They were identified based on their external and internal morphological characteristics to the species level by A. Feijoo (Universidad Tecnológica de Perreira, Colombia). Fresh biomass of all juvenile and adult earthworms was calculated for each of the two ecological groups represented (i.e., endogeic and epigeic species) according to criteria described by Bouché [68,69]. The biomass of one species, *Andiorrhinus* (*Turedrilus*) sp. (from here forward referred to simply as *Andiorrhinus* sp.), was measured separately, because of the high proportion of the total biomass of earthworms accounted for by this species in the *surales* ecosystem and because it does not fit neatly into either of the two ecological groups mentioned above. A two-way ANOVA was used to test the effects of the factors “site” (field sites) and “earthworm ecological group” (endogeics, epigeics and *Andiorrhinus* sp.) on variation in earthworm fresh biomass. Prior to this analysis, normality and homoscedasticity of data were confirmed using Shapiro-Wilk and Bartlett tests, respectively. A Wilcoxon rank-sum test was used to test the difference in earthworm biomass between mounds and inter-mounds, separately for each field site (α = 0.05).

### Analysis of soils

#### Soil properties

Sand, silt and clay contents (%) were measured on soil samples taken from 0–10 cm depth in each of the 60 sampling locations described in section 3. Analyses of soil texture were conducted following the method of Bouyoucos [70], the sample being dispersed in a sodium hexametaphosphate solution by overhead shaking. Texture class was determined using the USDA soil textural triangle [71]. Soil bulk density was measured using soil sampled from 0–10 cm below the soil surface on core samples taken with a 250 cm^3^ hollow cylinder pressed vertically into the soil at each sampling location. Following standard practice for measuring bulk density, which often shows high local-scale variation, three samples were taken at each location (see sampling design in section 3), giving a total of 135 samples in *surales* habitat (45 samples in each of Sites 1–3, 27 from mounds and 18 from inter-mound area in each field site). In Site 4, 45 samples were collected for determination of bulk density. Organic matter content (OM) was measured by the Loss On Ignition (LOI) method, with two replicates for each sampling location, giving a total number of 90 samples in *surales* habitat (30 samples per field site, 18 from mounds and 12 from inter-mound area in each field site). In Site 4 we collected 30 soil samples for analysis of OM content.

Soil profiles were described on two plots in each of the first two field sites along a trench cut one meter deep (from the soil surface of the inter-mound basin to the bottom) and four meters long. Each included a vertical profile of three mounds and the intervening basin. Mounds cut by the trench had an average (± SD) height of 15 ± 4.2 cm in Site 1 and of 37 ± 9.3 cm in Site 2.

X-ray analysis of the clay fraction was performed on two soil samples per soil profile in Sites 1–3, taken at 10 cm below the surface in the basin and from a mound, giving a total of 12 samples from *surales* habitat (4 samples per field site).

#### Soil structure

To characterize the contribution of biogenic macroaggregates to the composition of the uppermost 10 cm of the soil matrix, we adapted to the needs of our study the method proposed by Velasquez et al. [72], itself derived from the approach of Topoliantz et al. [73]. At each of the 60 sampling locations described in section 3, we collected from the soil surface (any litter was first removed) a monolith (5×5×10 cm) that was then air-dried for four days. Macroaggregates removed from these blocks were then characterized according to their apparent origin (aggregates associated with roots, earthworm-produced biostructures, aggregates and nest structures produced by social insects or aggregates resulting from physico-chemical processes) based on their morphology and size. In cases where it appeared that several actors had participated in the production of a macroaggregate, we classified the aggregate’s origin according to the ‘footprint’ that dominated the biogenic structure. Recently produced earthworm casts can be easily recognized, as they form clusters of smooth, round structures. Earthworm-produced biostructures also include burrows, easily recognized by their smooth, round walls, usually bearing closely spaced, parallel lines extending all around the burrow. Based on the results of Zangerlé et al. [74], we decided to consider as belonging to the burrow the soil that was located in the concentric layer of approximately 4 mm around each burrow. Macroaggregates associated with roots may have been directly produced by roots or may correspond to preexisting aggregates that were later colonized by plant roots. Physicogenic aggregates, produced by abiotic processes such as drying and wetting of the soil (often accompanied by activities of microorganisms, whose structural contributions are usually indiscriminable from those of abiotic processes without sophisticated analysis), are generally characterized by angular and sub-angular shapes. Physicogenic aggregates collected from soil of the inter-mound basin showed prismatic shapes and were on average larger than earthworm casts. They most likely resulted from drying and re-wetting processes occurring in the seasonally flooded basin. Physicogenic macroaggregates collected from mounds were more variable in size and shape. Some of these may have been derived from aged earthworm casts that had lost their smooth, rounded shapes. All remaining material, either unaggregated or forming aggregates smaller than 5 mm diameter (as determined by sieving), was considered to be non-macroaggregated soil. Each class of aggregate separated by this procedure (plus the fraction of non-macroaggregated soil) was air-dried over seven days and then weighed. A two-way MANOVA was used to test the effects of the factors “site” (field sites) and “habitat” (mound and inter-mound habitat) on variation in the five dependent variables (“earthworm”, “social insect”, “root”, “physicogenic” and “non-macroaggregated”) of the macroaggregate morphological data (α = 0.05).

## Results

### Spatial extent of *surales* landscapes

**Fig 2:**
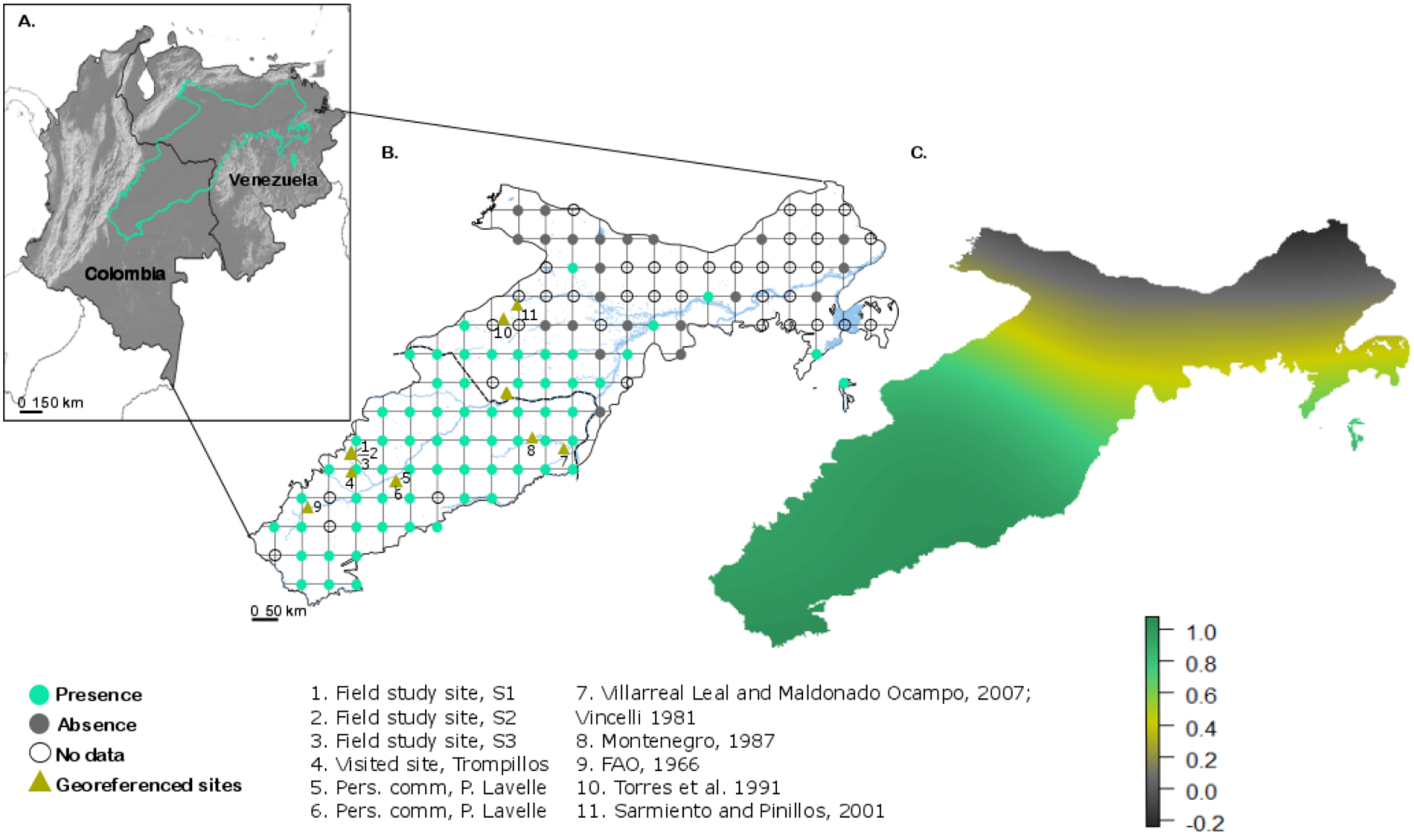
Spatial extent of *surales* landscapes. A. Spatial extent of the Orinoco Llanos. B. Observation grid of *surales* landforms used for the systematic survey and georeferenced *surales* sites from the literature and from personal communications. C. Estimated spatial extent of *surales* across the Orinoco Llanos. Values over 0.5 (green area) cover the area with the highest probabilities of observing these landforms.

The literature [6,8,75–78] allowed us to identify five *surales* sites (Fig 2A and 2B). Our fieldwork, and personal communications from collaborators, allowed identification of six additional sites. Based on our systematic survey of satellite imagery, we showed that of the 125 5-km-radius circles surveyed, 52% revealed patterned landforms similar to those in satellite images of our field sites and that were presumably *surales*. This analysis, together with the high-resolution aerial images taken in the field, revealed great variation in the shape of *surales*. They appeared as highly distinct round mounds and also in labyrinth patterns (see the GE visit). *Surales* were absent in 18% of the circles and satellite imagery of sufficient resolution was not available in 30.4% of the circles, mainly located in Venezuela (Fig 2B). All the sites surveyed in Colombia for which images of sufficient resolution were available showed *surales* landforms. The interpolated surface showed that the total area within which the probability of finding *surales* mounds within a pixel (defined in the methods section above) is superior to 0.5 represents 75,309 km^2^ (Fig 2C). This area corresponds to the part of the Orinoco Llanos that is most subject to seasonal flooding [34]. In our field work, we decided to focus on round-mound *surales*, leaving the more complex labyrinth patterns for later studies.

### Vegetation cover

Of the 500 total sampled points in the three sites covered by *surales*, mound and inter-mound habitats accounted respectively for 62.5% and 37.5% in Site 1, 74.5% and 25.5% in Site 2 and 82% and 18% in Site 3. Floristic composition of each transect and the relative cover abundance of each species on mounds and in inter-mounds are presented in S1 Table for the dry season and S2 Table for the rainy season. The tree species composition of Site 3 is presented in S3 Table.

#### Dry season

During the dry season, a total of 48 plant species were encountered in Site 1, and the same number of plant species were found in Site 2. The two sites had 15 species in common. Mound and inter-mound habitats shared 40% of the total number of plant species in Site 1 and 45% in Site 2. Whereas 52% of the total number of plant species recorded in Site 1 were found exclusively in the mound habitat, only 8% were found only in the inter-mound habitat. In Site 2, 32% of the total number of plant species were found only in the mounds, and 23% only in the inter-mounds. Species found in both habitats were usually markedly more abundant in one habitat than in the other (S1 Table).

**Fig 3:**
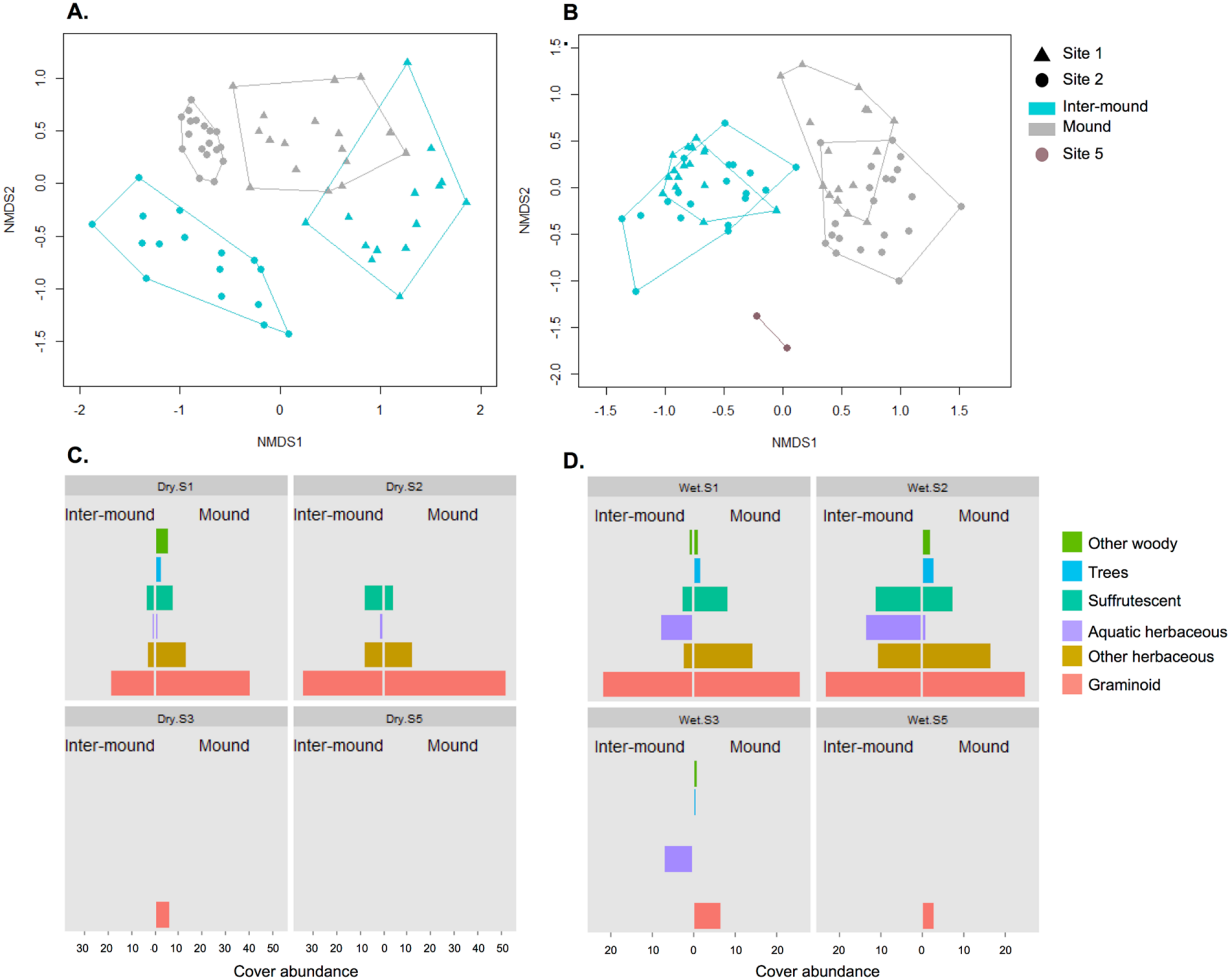
Plant community composition in *surales* landscapes and its seasonal variation. NMDS performed on the abundances of plant species collected during the dry (A) and the rainy (B) seasons. Plant life forms during the dry (C) and rainy (D) seasons. NB: For Site 3, where trees dominate on mounds, only the herbaceous substratum is represented here.

Of the total of 80 species encountered, 37 species accounted for 5% or more of the vegetation cover in at least one of the two habitats (mounds or inter-mound basins) in at least one of the two sites. The habitat type (mound, inter-mound) and the site respectively accounted for 14% and 22% of the variation in plant assemblage structure (relative cover abundance of each species; Adonis, *P* < 0.001 for both tests). The NMDS (stress value = 0.13, Fig 3A) showed that, for each of the habitat types, the difference in vegetation composition between sites was greater than that within sites, *i*.*e*., between transects. In Site 2, the ordination showed marked differences between the two habitat types, with vegetation on mounds and inter-mound basins forming well-differentiated clusters, with much less variation in vegetation composition on mounds than in inter-mound areas (Fig 3A). Species composition was less markedly different between habitat types in Site 1, where the two clusters overlapped. The distances among clusters revealed that the difference between the two sites is mainly explained by plant species composition in the inter-mound habitat. Regarding plant forms, the inter-mound habitat in Site 2 supported more suffrutescent, herbaceous and aquatic species compared to the inter-mound basin in Site 1 (Fig 3C). Mounds in Site 1 supported more diverse forms of plants, notably more woody species, compared to mounds in Site 2.

Vegetation in Site 3 differed greatly from that in Sites 1 and 2. In Site 3, the only species found in the lower stratum of vegetation on mounds was the sedge *Rhynchospora cephalotes*. This species, not encountered in any other of the sites studied, was recorded at each sampling point along the linear transect in Site 3. We identified eight tree species growing on mounds, ranging in height from two to seven meters (S3 Table).

#### Rainy season

During the rainy season, 44 plant species were encountered in Site 1, 53 in Site 2, and 10 in the *banco* site (Site 5). In Site 2, mound and inter-mound habitats shared 45% of the number of species recorded (S2 Table). About 32% of species were recorded only on mounds, and 23% were recorded only in inter-mound habitat, proportions which were comparable to those found in the dry season. The main seasonal changes occurred in Site 1, where the percentage of species shared between habitats decreased from 40% during the dry season to 25% during the rainy season. This change resulted from a dramatic increase, from 8% during the dry season to 32% during the rainy season, of plant species only recorded in inter-mound habitat. The *banco* shared four plant species with the whole set of plants encountered in Sites 1 and 2.

Of the total of 67 species recorded in Sites 1, 2 and 5, 37 attained 5% or more of the vegetation cover in at least one of these three sites. Results of the non-parametric MANOVA revealed that habitat type (mound, inter-mound) accounted for 34%, and site for 12%, of the variation in the plant assemblage structure (*P* < 0.001 for both tests). In contrast to our results in the dry season, the NMDS (stress value = 0.15, Fig 3B) showed a greater differentiation in vegetation composition between mounds and inter-mounds compared to the differentiation between sites. The NMDS also showed that the differentiation among sites was mainly due to i) differences in species composition on mounds, even though the diversity of plant life forms was similar (Fig 3D), and ii) to the marked differences between the *surales* sites (Sites 1, 2) and Site 5. Regarding plant life forms, herbaceous aquatic plants were present in the inter-mound areas of both Sites 1 and 2, but were more abundant in Site 2, as were other herbaceous species (excluding graminoids) and suffrutescent plants. Vegetation of the Site 5 featured very low diversity of plant life forms, composed almost only of graminoids.

In Site 3, during the rainy season eight tree species were recorded on mounds, ranging in height from two to nine meters. Using the point-intercept method, seven plant species were recorded, of which only one species was shared with Site 1 (*Davila nitida*) and one with Site 2 (*Miconia* sp.).

#### Dry and rainy seasons compared

Vegetation structure, composition and diversity underwent substantial changes from the dry to the rainy season in Sites 1 and 2. First, in both sites the density of plants in inter-mound habitat increased, whereas it decreased on mounds. This trend is reflected by the total number of plants contacted in the line transects (S1 and S2 Tables). The species composition was also remarkably different between the dry and the rainy seasons. In the mounds, only 14% of the plant species were found in Site 1 in both seasons, and 9% in Site 2. In the inter-mound basins, only 4% of the plant species were found in site 1 in both seasons, and 9% in site 2. Graminoids (Poaceae, Cyperaceae, Eriocaulaceae) decreased in abundance in the rainy season, while herbaceous aquatic species increased in abundance (Fig 3D), with new species appearing, such as *Nymphaea* cf. *blanda* and several species of *Utricularia* (S2 Table). Whereas individuals of most species present in these sites are perennial, above-ground parts of most of the herbaceous and suffrutescent species are strongly reduced in the unfavorable season. As in Sites 1 and 2, herbaceous aquatic species appeared in the inter-mound basin during the rainy season in Site 3.

#### The distribution of panicoid grasses

Among the taxa abundantly represented in our vegetation samples, the subfamily Panicoideae of the grass family (Poaceae) is the only one to possess phytoliths that are quasi-diagnostic (given the absence in our sites of other grass subfamilies that have similar phytoliths (e.g., Bambusoideae: [64]), thereby permitting direct comparison of distribution of this taxon in present-day vegetation and occurrence of its phytoliths in soils. We thus tabulated separately the representation of Panicoideae in our vegetation samples, to facilitate comparison with soil phytolith composition. Panicoid grasses were abundant in Sites 1 and 2, but absent from Site 3. In Sites 1 and 2, panicoids were 2 to 15 times more abundant on mounds than in inter-mound basins, depending on site and season. In Site 1, nine species of panicoid grasses were encountered. Their collective contribution to cover abundance on mounds was 30.8% in the dry season and 42.0% in the rainy season. In inter-mound basin habitat, the contribution of panicoid grasses to cover abundance was very similar in the dry and the rainy seasons (10.1 and 10.4%, respectively). In Site 2, 13 species of panicoid grasses were encountered. They accounted for 56.5% of cover abundance on mounds in the dry season and 37.0% in the rainy season. In inter-mound basins, they accounted for 27.6% of cover abundance in the dry season, but only 2.4% in the rainy season. To summarize, species richness of panicoid grasses was higher in Site 2 than in Site 1, as was their contribution to cover abundance in the dry season. The greatest proportional difference in cover abundance of panicoids (15-fold) was in Site 2 during the rainy season, where six species of panicoids were recorded on mounds but only one (*Eriochloa* sp.) was rarely recorded in the deep basins of this site.

### Phytoliths

**Fig 4:**
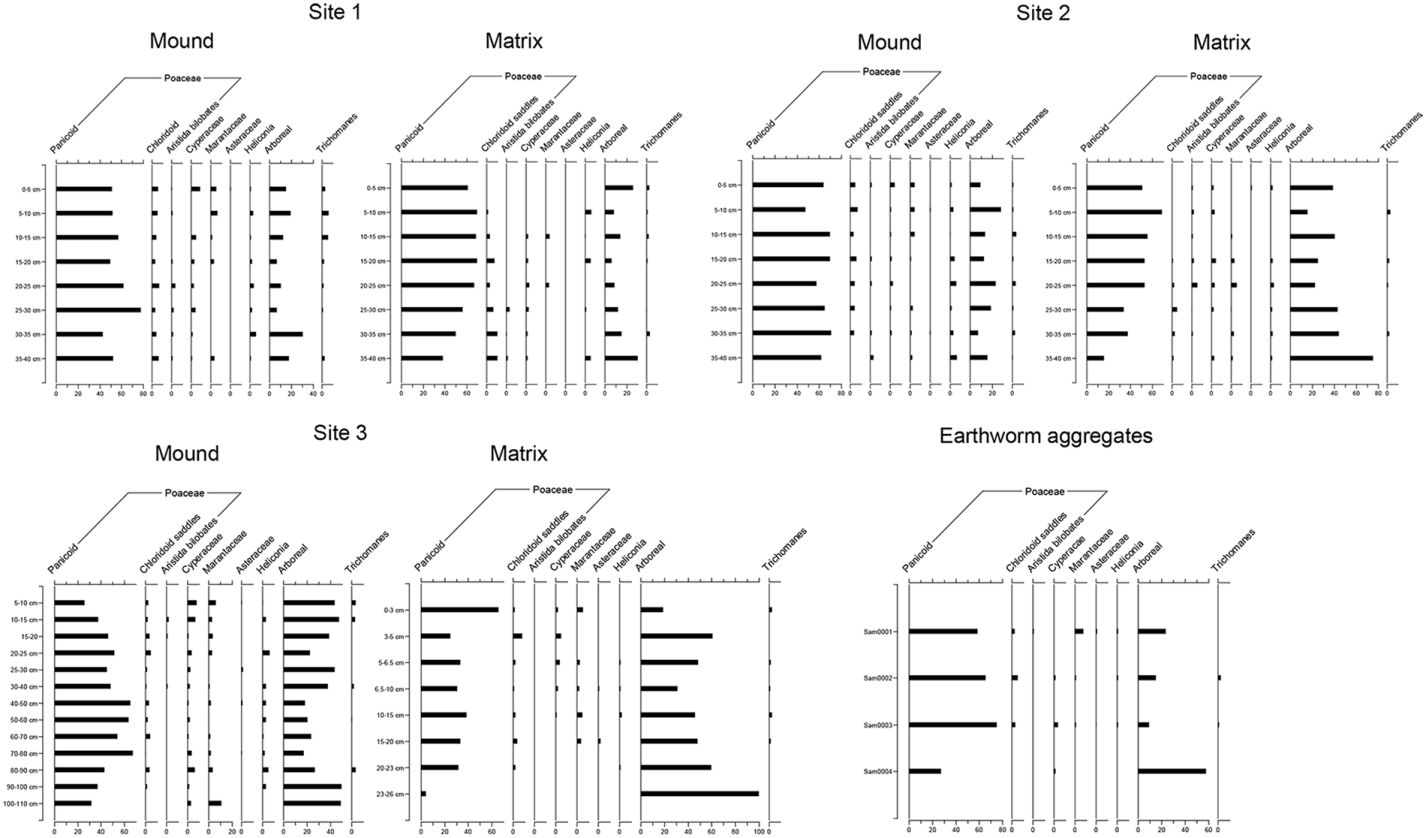
Summary diagram of phytolith morphotype composition in soils of Sites 1–3 and in earthworm casts. Horizontal bars represent percentages of phytolith morphotypes for each soil layer.

Overall, the phytolith record showed no clearly discernible patterns. Not surprisingly, the proportion of arboreal phytolith morphotypes tended to be greatest in Site 3, the only site where trees were present. However, few other patterns were found. Surprisingly, there was little relation between composition of current vegetation and that of phytoliths in surface soil horizons. The great variation between mound and inter-mound habitats in each site, and in each of these habitat types across sites, was by and large not mirrored in the phytolith composition of surface soil. Phytolith morphotypes characteristic of panicoid grasses illustrate this point particularly clearly. In Sites 1 and 2, although grasses of this taxon were 2–15 times more abundant on mounds than in inter-mound basins, depending on site and season, no marked difference was apparent in the representation of phytolith morphotypes characteristic of panicoids in surface soils between these two habitats in either site (Fig 4). Variation in phytolith composition with depth in soil also showed no apparent pattern related to habitat (mound and inter-mound) or site. Finally, no phytolith morphotypes typical of crop plants such as maize (*Zea mays*), manioc (*Manihot esculenta*) or arrowroot (*Maranta arundinacea*) were recovered in the samples analyzed. There is thus no indication that these mounds of natural origin were ever used as agricultural raised fields.

The composition of phytoliths found in earthworm casts was similar, in terms of diversity and relative abundances, to that found in soils of the site (Site 1) from which they were collected. Phytoliths characteristic of panicoid grasses accounted for 27–59%, followed by globular psilate phytoliths (9–26%) and silicified anticlinal polyhedral epidermal cells (9–27%). As mentioned above, these last two morphotypes have very low taxonomic resolution, being produced by both herbaceous and woody dicots, as well as by herbaceous monocots [79]. Phytolith morphotypes characteristic of woody taxa were infrequent (5–6%), as they are in soils of this site (Fig 4).

### Species richness and biomasses of earthworms

**Fig 5:**
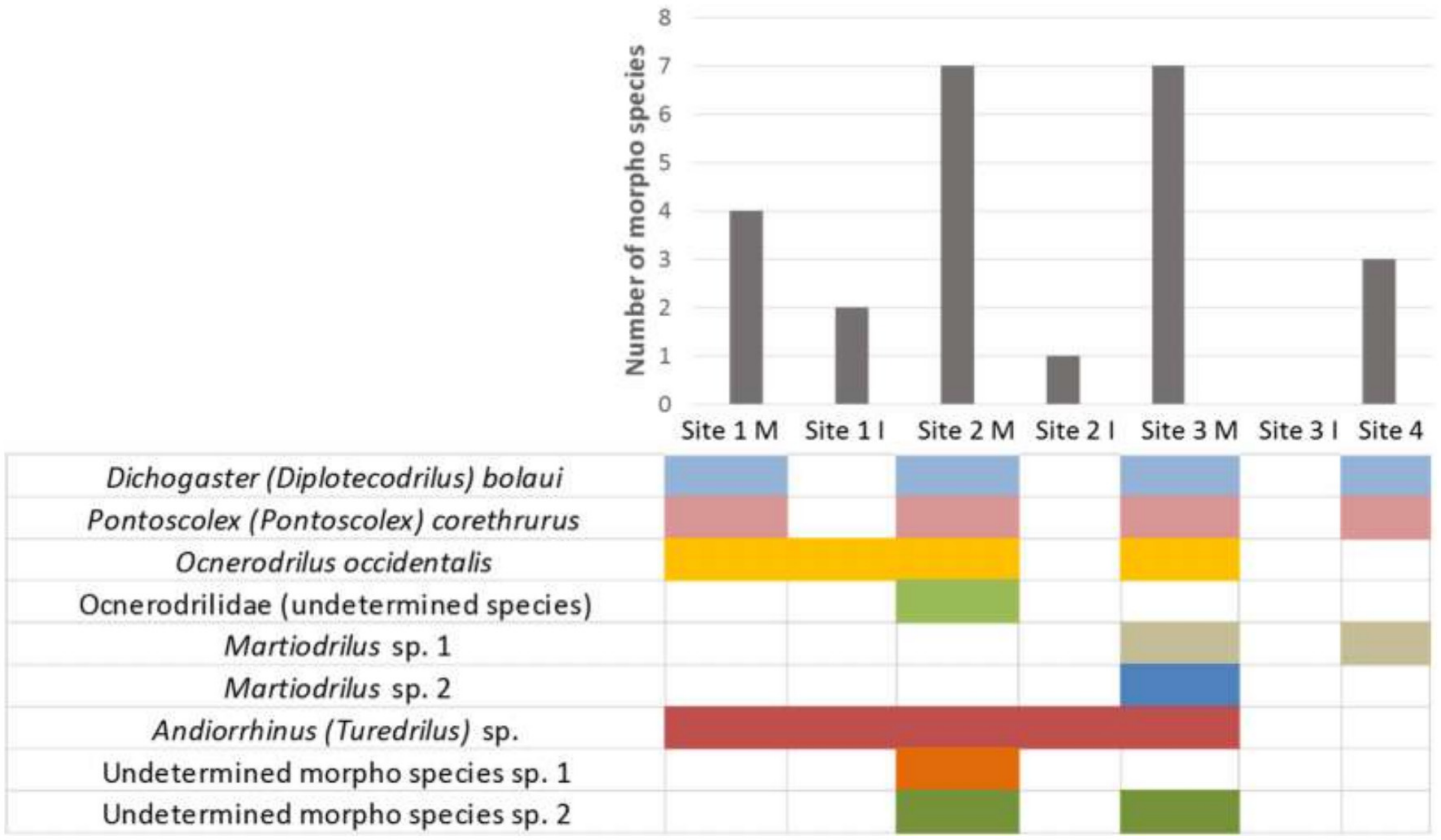
Earthworm species diversity and composition in mound and inter-mound habitats in *surales* field sites (Sites 1–3) and the control (banco) site (Site 4). M: Mounds, I: Inter-mound basin.

We identified a total of nine morphospecies of earthworms in the *surales* field sites. Site 1 had the lowest species richness, with four species, all of which were also encountered in the two other sites: *Andiorrhinus* sp., which does not fit neatly into ecological groups, and three endogeic species (*Dichogaster* [*Diplotecodrilus*] *bolaui*, *Pontoscolex* [*Pontoscolex*] *corethrurus* and *Ocnerodrilus occidentalis*). Site 2 harbored, in addition to these four, three additional endogeic species (*Ocnerodrilus occidentalis*, an undetermined morphospecies of the family Ocnerodrilidae and two morphospecies undetermined at the generic level). Site 3 also harbored seven species: the four species found in both other sites, one of the two undetermined morphospecies found in Site 2 and two epigeic species (two undetermined species of *Martiodrilus*). Epigeic species were thus found only in the *surales* site with the largest mounds and best-drained mound-top soils. In all three sites, earthworm species richness was higher on mounds than in inter-mound habitat, and Site 3 was characterized by a complete absence of earthworms in inter-mound habitat (Fig 5).

**Fig 6:**
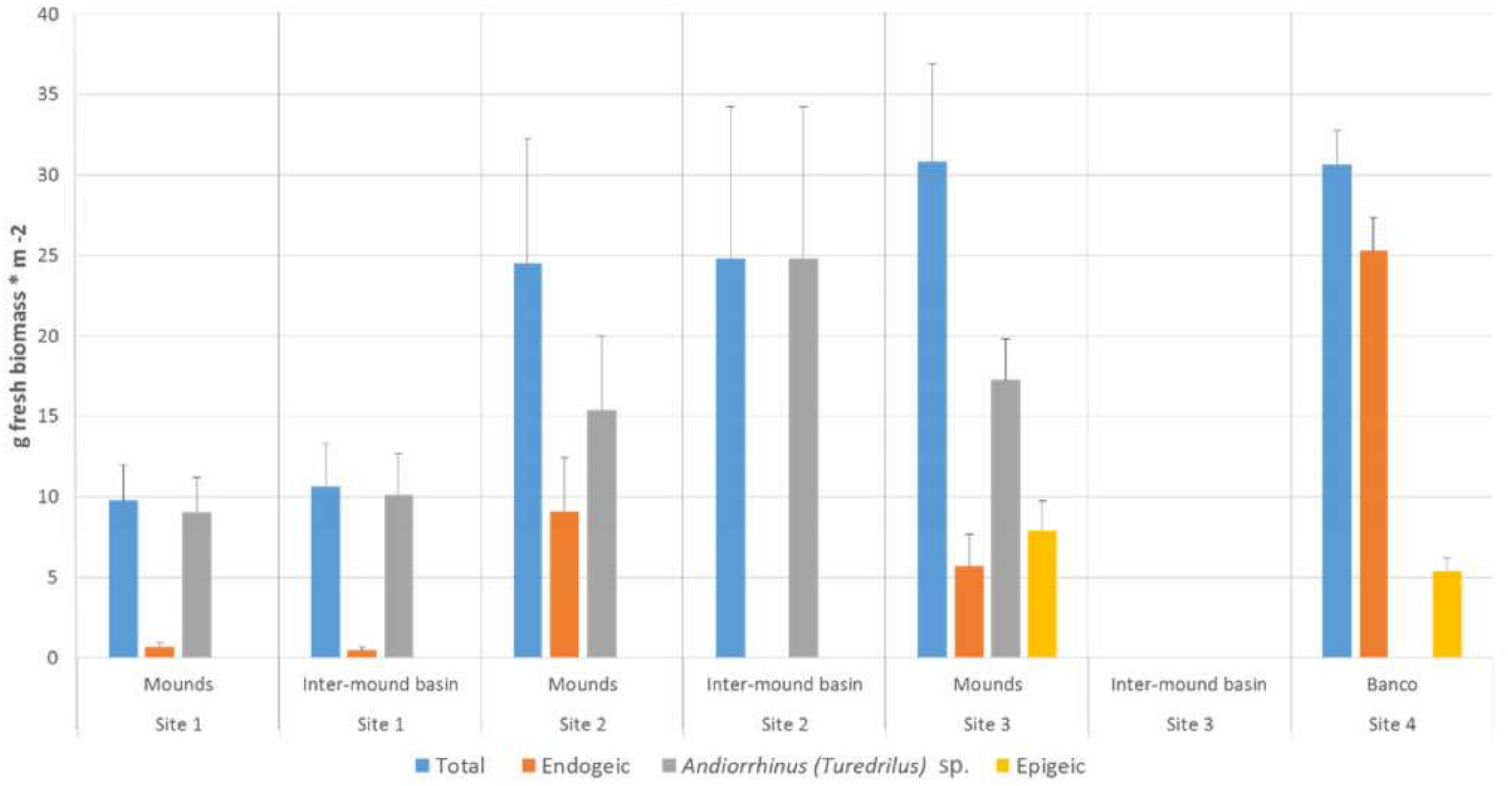
Earthworm biomass for ecological groups. Fresh earthworm biomass (g*m^-2^) for ecological groups (endogeics and epigeics), *Andiorrhinus* sp. and the total of all species in mound and inter-mound habitats in *surales* field sites (Sites 1–3) and the control site (Site 4).

In terms of biomass, the single species *Andiorrhinus* sp. was predominant in all three *surales* sites (Fig 6), particularly in Site 1, where it accounted for over 92.9% of total earthworm biomass in both mound and inter-mound habitats. In Site 2, this species attained even higher biomass than in Site 1, particularly in inter-mound habitat, where it was the only earthworm present. In Site 3 it (like all other earthworms) was encountered only in mound soils. The biomass of endogeic earthworms was very low in both mound and inter-mound habitats in Site 1 and reached somewhat higher values in Sites 2 and 3, where they were present only in mound soils. As noted above, epigeic earthworms occurred only in Site 3, where they were restricted to mound soils and found at biomass similar to that of endogeic species.

The most striking feature of earthworm communities in Site 4, the *banco*, was the complete absence of *Andiorrhinus* sp. Only three species were encountered in this site, two endogeic and one epigeic species (Fig 6), but total earthworm biomass (dominated by endogeics) was comparable to the highest values found in the *surales* sites (Fig 6).

### Soil properties and soil structure

#### Description of soil profiles

The elevated parts of the mounds were composed of a single soil horizon (A1 horizon), gray in color with brown mottling and containing abundant earthworm burrows and roots. The horizon at the surface of the inter-mound basin (and underlying the mounds) was noted A2. In both sites, this horizon was 15–20 cm deep, light gray in color and characterized by the presence of very coarse subangular blocky structures (aggregates > 80 mm) according to the FAO Guidelines for Soil Description [80]. In basins, the A2 horizon showed a few large vertical earthworm burrows with yellowish brown mottles on their walls. In the basin, the bottom of the A2 horizon was marked by a dark layer 1–4 cm thick. Beneath this layer (and beneath the A2 horizon in both basin and mounds), the Bg horizon was characterized by a combination of significant iron or manganese concretions and a light gray to brownish gray matrix with some orange mottling, typical of soils with *gleyic* properties [81]. This Bg horizon thus appears to be strongly influenced by the fluctuating level of groundwater in soils. A 5-cm deep gravel lens was located between horizons A2 and Bg in the inter-mound basins of Site 1. Texture of the A horizon of both mounds and basin was silt loam in Site 1 and silt-clay in Site 2 (Table 1). Soils had relatively low OM contents (Site 1: 2.0 ± 0.3% in mounds and 2.0 ± 0.1% in basins; Site 2: 2.8 ± 0.2% in mounds and 2.1 ± 0.6% in basins) and were characterized by a high bulk density (mean value 1.6 ± 0.01 g x cm^-3^ in mounds of both sites and 1.7 ± 0.03 g x cm^-3^ in basins of Site 1 and 1.8 ± 0.02 g x cm^-3^ in basins of Site 2 (Table 1). All soils were somewhat acid; pH_water_ values varied between 5 and 5.1 in both mounds and basin (Table 1).

**Table 1:** Main soil characteristics in the field sites.

		CO (%)	*SD*	OM (%)	*SD*	pH	Bulk density	*SD*	Clay (%)	*SD*	Silt (%)	*SD*	Sand (%)	*SD*
Site 1	Mound	1.2	*0*.*2*	2	*0*.*3*	5	1.6	*0*.*01*	17.8	*3*.*7*	63	*2*.*6*	19.2	*2*.*6*
Site 1	Basin	1.2	*0*	2	*0*.*1*	5.1	1.7	*0*.*03*	16.8	*2*.*2*	61.8	*1*.*3*	21.3	*2*.*4*
Site 2	Mound	1.6	*0*.*1*	2.8	*0*.*2*	5.1	1.6	*0*.*01*	44.1	*8*.*3*	41.6	*4*.*6*	14.7	*5*.*5*
Site 2	Basin	1.2	*0*.*3*	2.1	*0*.*6*	5.1	1.8	*0*.*02*	43	*8*.*7*	42	*4*.*4*	15.3	*8*.*8*
Site 3	Mound	3.6	*0*.*1*	6.2	*0*.*1*	4.6	1.5	*0*.*01*	15	*7*.*5*	44	*10*.*0*	41.5	*3*.*2*
Site 3	Basin	2.6	*0*.*3*	4.5	*0*.*2*	4.6	2.1	*0*.*03*	11.7	*3*.*4*	51.3	*2*.*2*	37.7	*2*.*4*
Site 4 (Banco)		2.2	*0*.*3*	3.8	*0*.*6*	6	1.3	*0*.*01*	6.7	*2*.*8*	18	*3*.*0*	75.3	*5*.*2*

At Site 3, soil profiles were described in two trenches, each in a single mound, cut 90 cm deep from the top of the mound and approximately 2 m long. Each profile in a mound was characterized by a single A1 horizon of loamy soil with a very homogeneous dark gray color. Soils had relatively high OM contents (6.2 ± 0.1%); average bulk density was 1.5 ± 4.6 g x cm^-3^ (Table 1). During the dry season the inter-mound basin of Site 3 was still covered by approximately 70–90 cm of water. Cores of the basin soil thus had to be collected using an auger (diameter 8 cm, depth 1 m) to characterize the horizon. The first 5 cm below the soil surface were characterized by loamy dark brown material. The bulk density of this A2 horizon was very high (mean value 2.1 ± 0.03 g x cm^-3^), as was OM content (4.5 ± 0.2%) (Table 1). The Bg horizon was gray in color with yellow to dark brown mottles, indicating a strong influence of groundwater movement (*gleyic* properties), as observed in the Bg horizon of Sites 1 and 2. The walls of the mounds in this site were not vertical: beneath the mound’s rounded dome, walls were concave. Mounds were thus “mushroom-shaped” (see Fig 1). Large areas of the walls of the mounds were covered by large, freshly produced earthworm casts. Soils in Site 3 were slightly more acid (pH 4.6 in mound and basin) than in the other two field sites.

Description of soil profiles and analyses of soils of the three *surales* sites (Sites 1–3), allowed us to classify the soil as Fluvic Gleysols (Siltic, Uterquic, Epivermic), according to the World Reference Base for Soil Resources [81].

Soils of Site 4 (*banco*) showed a single horizon with a sandy texture. Soils had relatively high OM contents (3.8 ± 0.6%) and were characterized by an average bulk density of 1.3 ± 0.01 g x cm^-3^ (Table 1). Soils in this site were somewhat less acid (pH 6.0) than in the *surales* sites (Table 1). Soil properties were not measured for Site 5 (natural levee). However, according to Goosen [32], who conducted a soil inventory in the area of San Luis de Palenque, soils of the natural levees are all characterized by texture ranging from fine sand to sandy loam.

X-ray analyses of the clay fraction showed the predominance of kaolinite (> 50%) and quartz (15–30%). It is noteworthy that quantities of 2:1 clay minerals (< 15% of the clay fraction) were insufficient to generate gilgai microtopography by shrinking/swelling dynamics typical of Vertisols. Furthermore, we observed neither a Vertic horizon nor shrink-swell cracks at the soil surface of Sites 1–3, excluding any scenario for genesis of these landscapes based on purely physical factors.

#### Soil structure

**Fig 7:**
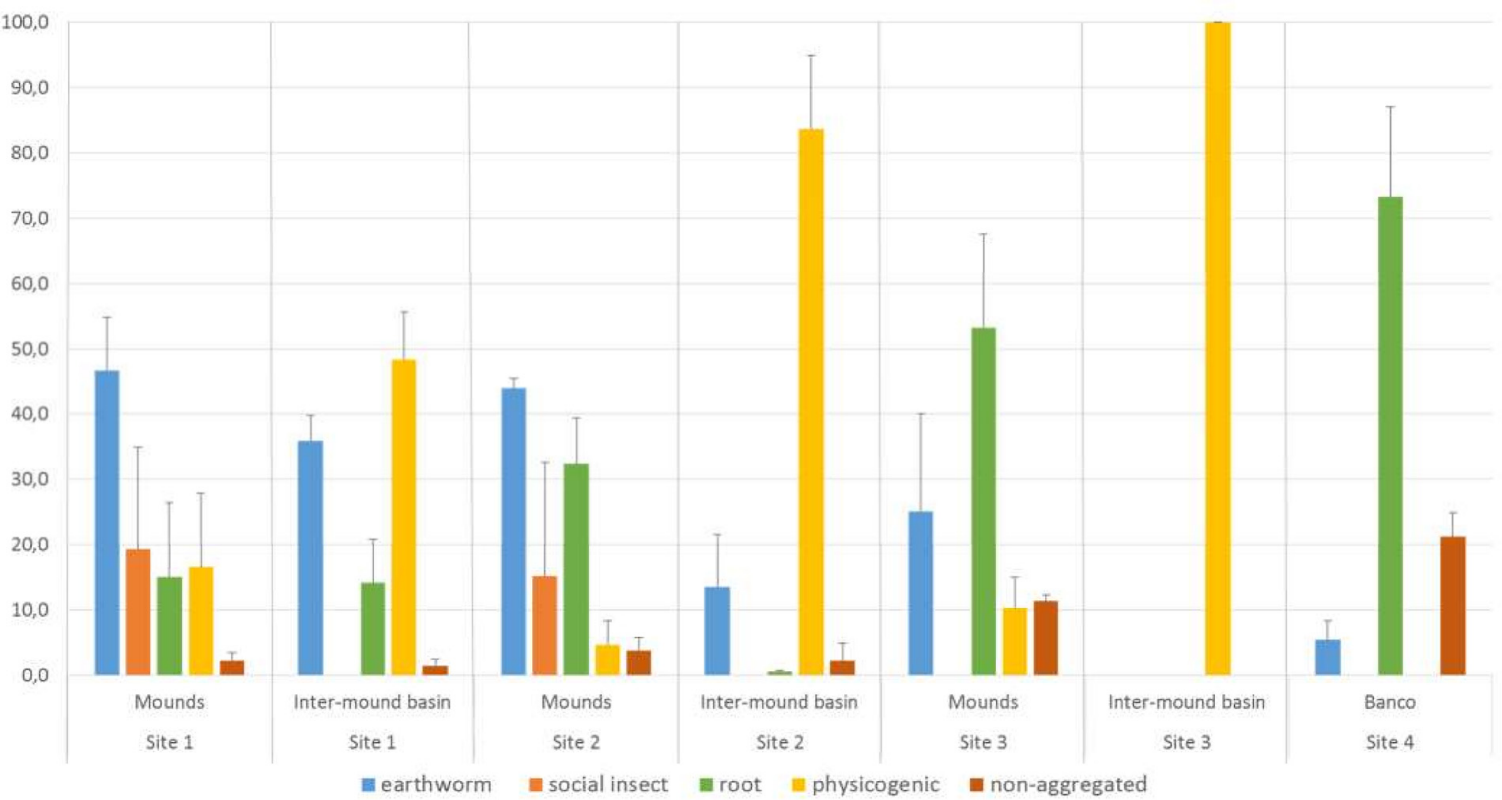
Composition of the surface soil matrix. Soil monoliths were extracted from the uppermost 10 cm of the soil profile in mound and inter-mound habitats in *surales* field sites (Sites 1–3) and the banco site (Site 4). Results are expressed as percentages of the total soil dry mass (g).

Morphological assessment of macroaggregates collected from the upper 10 cm of soil in *surales* field sites showed large differences in the proportions of macroaggregates of different origins between the two kinds of habitats, mounds and inter-mound basin (Fig 7). In all three sites with *surales*, soils of the inter-mound basin were strongly dominated by macroaggregates of physicogenic (as opposed to biogenic) origin, and this dominance increased from Site 1 to Site 3. Physicogenic aggregates accounted for 48.4 ± 7.2% (mean ± SD) of total soil mass in Site 1, 83.7 ± 11.2% in Site 2 and 100 ± 0.0% in Site 3. In contrast, mound soils were dominated by various types of macroaggregates formed by organisms. Biostructures produced by earthworms comprised most of the mass of macroaggregates in mound soils in Sites 1 and 2, accounting for 46.7 ± 8.1% and 44.0 ± 1.5% of total soil mass, respectively. These two proportions were not significantly different from each other (two-way MANOVA, *P* > 0.05). In Site 3, biostructures identifiable as having been recently produced by earthworms comprised a smaller proportion of the total mass of mound soils, 25.1 ± 14.9%. In all three sites, earthworm-produced biostructures accounted for smaller proportions of total soil mass in the inter-mound basin compared to mounds, with means of 35.9 ± 3.9% in Site 1, 13.5 ± 8.1% in Site 2 and 0% in Site 3. The proportional difference between mound and inter-mound in the contribution of earthworm-produced biostructures to soil structure was greater in Site 2 than in site 1, and greatest (infinity) in Site 3. This difference was not significant in Site 1 (two-way MANOVA, *P* > 0.05) but was highly significant in Sites 2 and 3 (two-way MANOVA, *P* < 0.001 in both sites). Macroaggregates (including nest structures) produced by social insects were present in mound soils in Sites 1 and 2, but completely absent from inter-mound soils in all three sites. As the proportion of soil mass constituted by biostructures produced by earthworms and by social insects decreased in mound soils from Site 1 to Site 2 to Site 3, the proportion constituted by root-associated macroaggregates simultaneously increased, from 15.1 ± 11.3% of total soil mass in Site 1, to 32.4 ± 7.0% in Site 2 and 53.2 ± 14.4% in Site 3.

Soil of Site 4 (*banco*) was dominated by root-associated macroaggregates, which accounted for a mean of 73.3 ± 13.7% of total soil mass. Earthworm-produced aggregates accounted for 5.4 ± 2.9%, and 21.3 ± 3.6% of soil was non-aggregated. Physicogenic macroaggregates were not observed in these sandy, relatively well-drained soils.

## Discussion

We estimated that *surales* landscapes occur within an area comprising 75,309 km^2^ of the Orinoco Llanos. An overview of the extent and the diversity of these spectacular landscapes is provided by a Google Earth^™^ visit (https://mycore.core-cloud.net/public.php?service=files&t=e01a65c5769726d35c4f005a13c46502). As satellite images of sufficient resolution for Venezuela (and of improved resolution for Colombia) become publicly available, our estimate could be revised. An important corpus of literature acknowledges the effects of such earth-mound landscapes on species diversity and spatial distribution and on ecosystem processes. This first tangible evidence of the great spatial extent of these landscapes presented here reveals that *surales* can no longer be ignored in work on the ecology of this ecoregion. But these ecosystems are under threat from industrial agriculture, and are being leveled to make way for highly intensified commercial production of rice [82,83], with the risk that they may disappear before they can be understood. Recognition of the widespread presence of *surales* in the area and better understanding of their ecology could spur efforts to conserve these landscapes, including research on how they can be managed for the benefit of both biodiversity and human well-being. With this study, we provide the first pieces of the puzzle to help us understand the processes that create *surales*, determine their development and mediate their effects on biodiversity.

### The key role of bioturbation by earthworms in formation of *surales*

In this paper, we present several lines of evidence supporting the contention that bioturbation by earthworms—and possibly other soil engineers—explains the formation of *surales* landscapes.

**Fig 8:**
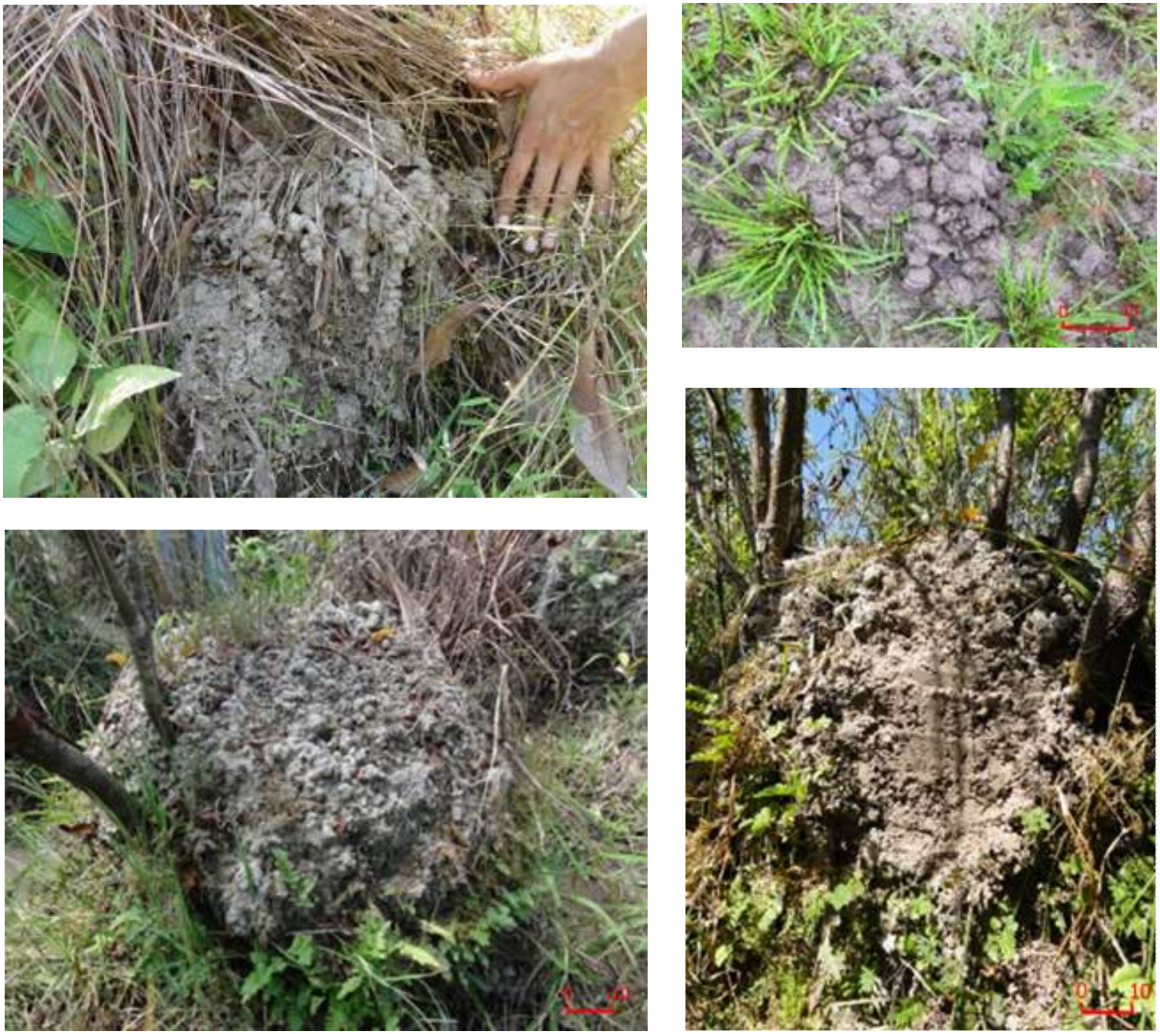
*Surales* mounds covered by casts at Site 3. Photos Anne Zangerlé 2012.

First, our results are consistent with previous observations showing that mounds consist largely of earthworm casts, with freshly produced casts literally covering the mounds in the *surales* sites studied (Fig 8). Although earthworms were collected in soils of the inter-mound basins and high burrow densities were observed in them, inter-mound soils contained very small amounts of casts. This result suggests that earthworms forage in the basin and deposit the ingested soil mostly on mounds. Transport of soil to mounds is a dynamic process, with casts being repeatedly deposited in the same locations by individual earthworms and with numerous individual earthworms contributing to the formation of large mounds (Fig 8). The occurrence of a “stone line” in the inter-mound area of Site 1 strengthens this hypothesis. Since earthworms cannot ingest and displace coarse particles such as gravel from the basin to the mounds, gravel tends to accumulate in the basin at the base of the “biomantle” [84–86].

Secondly, patterns in soil phytolith composition in the three sites indicate very effective mixing of soil both horizontally (between mound and inter-mound habitats) and vertically (over soil profiles) by bioturbating soil engineers, most importantly earthworms. In principle, phytolith composition of soil surface layers should reflect the current vegetation cover. Because leaves and other organic matter containing phytoliths usually decompose where they fall, rather than being moved, soil phytoliths reflect vegetation composition at a very local scale (in contrast to pollen) and thus reflect the variation of vegetation cover across habitats [87,88]. Given the marked difference in vegetation between mound and inter-mound habitats (e.g., in the cover abundance of panicoid grasses), we would also have expected, in the absence of very high rates of bioturbation, differences in soil phytolith composition near the surface in these two habitats. Such differences were observed in vestigial raised-field landscapes in French Guiana, even though bioturbation was active [89] and despite the fact that differences in present-day vegetation of mound and basin were less marked than those we observed in the *surales* sites [5]. However, in *surales* landscapes, we found no striking differences in phytolith composition near the surface in soils of mounds and of the adjacent basin.

Thorough mixing of soil by bioturbating soil engineers could also explain the absence of marked vertical patterns of variation in phytolith composition with depth in soil, in a given habitat. Vegetation change over time—which we believe has been substantial with the development of *surales* landscapes (further discussed below)—is often marked by variation in phytolith composition with depth in soil [57,79]. With regards to Sites 1 and 2, if (as we postulate) *surales* grew vertically in a flooded plain to rise above the flood water level, increasing in size from towers to mounds, creating drier platforms colonized by vegetation that cannot thrive under waterlogged conditions (such as panicoid grasses and arboreal taxa), in the absence of bioturbation we should expect an increase in phytoliths of these taxa near the surface and in the upper levels of *surales* soils, and in turn a decrease in the proportion of wetland-adapted taxa (e.g., Cyperaceae, Marantaceae). We were anticipating a pattern similar to that we had already documented in vestiges of human-made raised fields in French Guiana, where a clear decrease in wetland taxa towards the upper levels of raised fields was apparent, with a concomitant increase in panicoid grasses [5, 79,90]. However, this was not the case. For example, in the mound phytolith profile in Site 1, panicoid grasses showed a decreasing trend toward the surface and in the mound phytolith profile in Site 2, they fluctuated with no clear increasing trend. Similarly, at Site 3, if bioturbation were not significant, we would have expected the phytolith record to show an increase in arboreal taxa towards the surface, at the expense of wetland taxa. However, arboreal taxa fluctuate throughout the profile and wetland taxa tend to increase toward the surface. Although panicoid grasses are lacking from present-day vegetation in Site 3, their phytoliths are abundant in its soils, occurring throughout the depth profile. This not only testifies to the former presence of panicoid grasses in the site, but is also a further indicator of very active bioturbation.

Several processes can modify the distribution of phytoliths in soil after their deposition [88,91,92]. However, bioturbation is the only process likely to be capable of effecting the massive translocation of phytoliths, both vertical and horizontal, suggested by our results. Bioturbation is acknowledged to be an important mechanism influencing the distribution of phytoliths in soils [92,93]. Termite galleries, for example, can lead to the existence side by side in the same soil horizon of two distinct phytolith pools [88]. However, such effects pale in comparison to those suggested for our *surales* sites, where phytolith distribution is virtually homogenized across two very different habitats and over soil profiles. In fact, we know of no other site where phytolith composition shows so little variation with soil depth, and where phytolith composition near the soil surface shows so little correspondence with current vegetation cover, as in the *surales* sites studied here.

In conclusion, the relative homogeneity of soil phytolith composition across habitats, sites and depths in soil is consistent with the hypothesis that the *surales* soil matrix is the result of the accumulation of aggregates that have been subjected to high rates of bioturbation, with soil being moved both vertically and horizontally.

### One earthworm species appears to initiate mound formation

**Fig 9:**
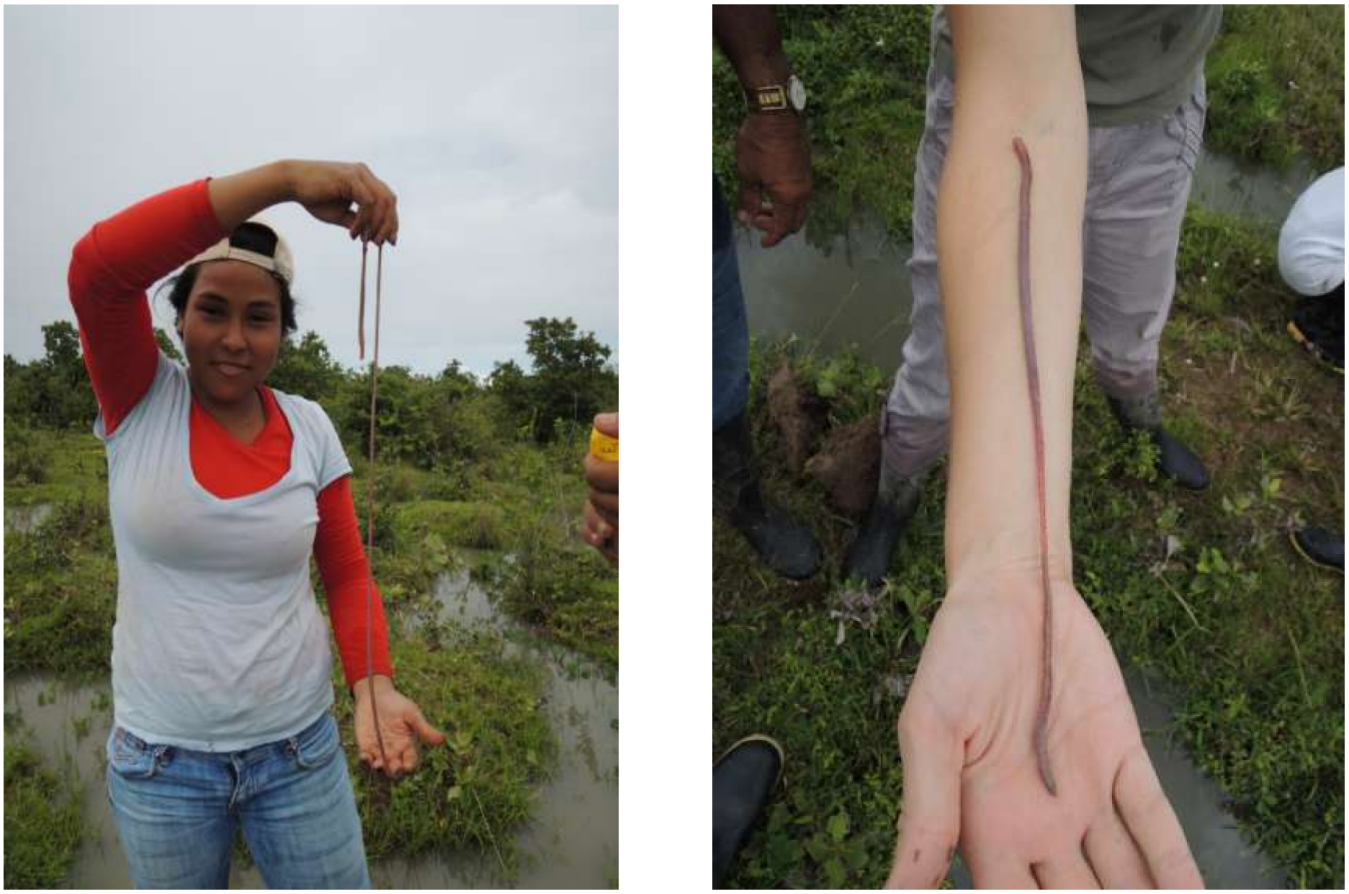
Pre-adult specimen of *Andiorrhinus* sp. Photos Doyle McKey 2012.

Most earthworm casts found on the soil surface and in the soil matrix of *surales* mounds were unusually large, with a mean diameter of 5.3 ± 3.0 cm, indicating that most casts were produced by very large earthworms. One earthworm species, *Andiorrhinus* sp., the largest species in these sites (pre-adult individuals collected in this study reached up to 1 m in length), accounted for most of the total earthworm biomass in all mound soils (Fig 9). The hypothesis that this species is the builder of *surales* mounds is supported by three other findings. First, this species was dominant in the smallest mounds (92.9% of the biomass in Site 1), indicating that this species is responsible for initiating the formation of mounds. Secondly, with the exception of *Ocnerodrilus occidentalis*, found at very low biomass density in Site 1, *Andiorrhinus* sp. is virtually the only earthworm found in the waterlogged inter-mound soils, and thus the only one whose foraging activities could move soil from inter-mound areas to mounds, where all its casts are found. Third, this species appears to be restricted to the seasonally flooded soils in which *surales* appear, being absent from the never-flooded *banco* site (Site 4).

### This earthworm species drives spatial self-organization in *surales* landscapes

**Fig 10:**
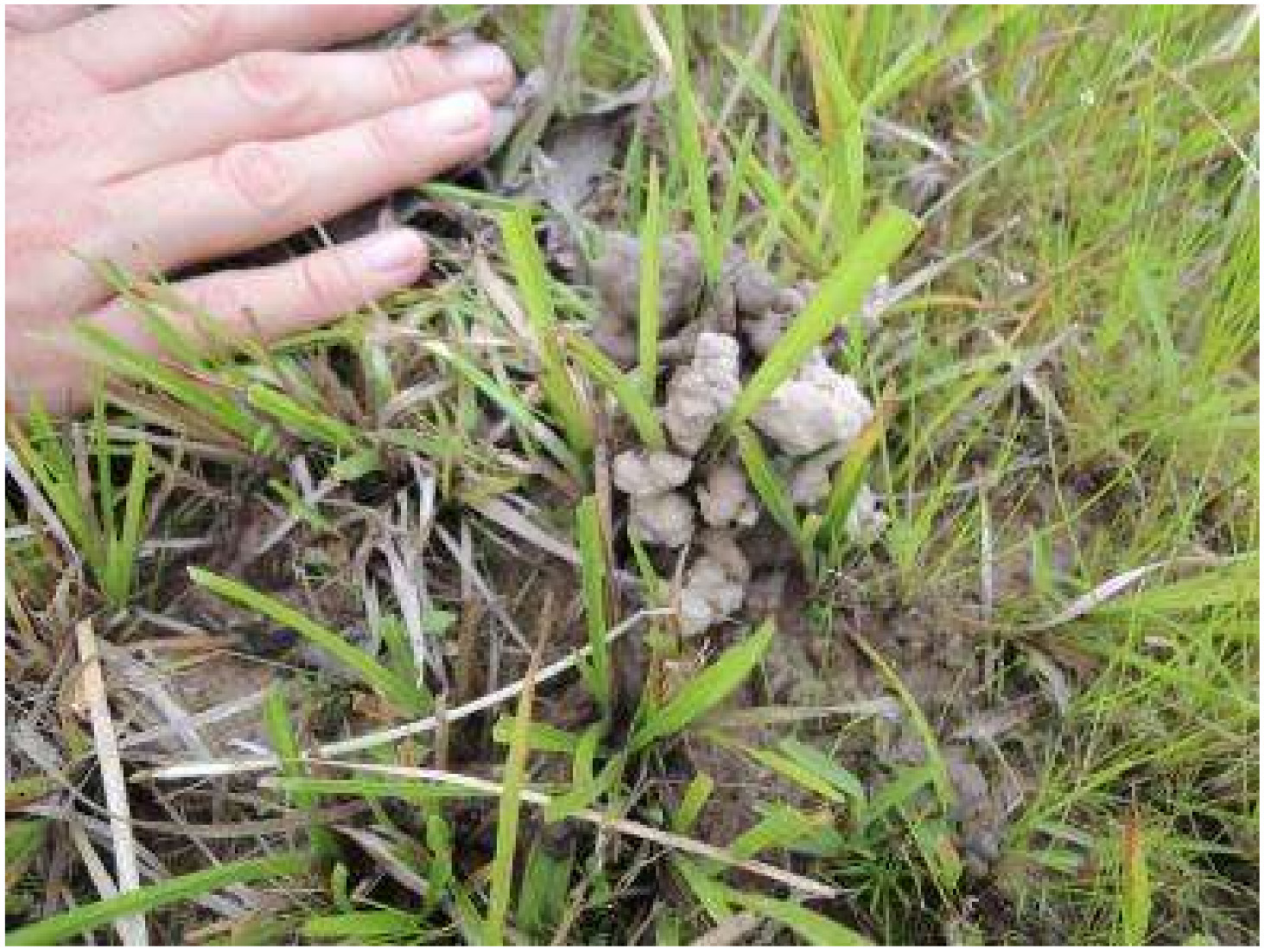
Accumulation of a freshly emitted cast (still moist) and an older cast (dried) over the opening of a burrow of *Andiorrhinus* sp. Photos Doyle McKey 2012.

**Fig 11:**
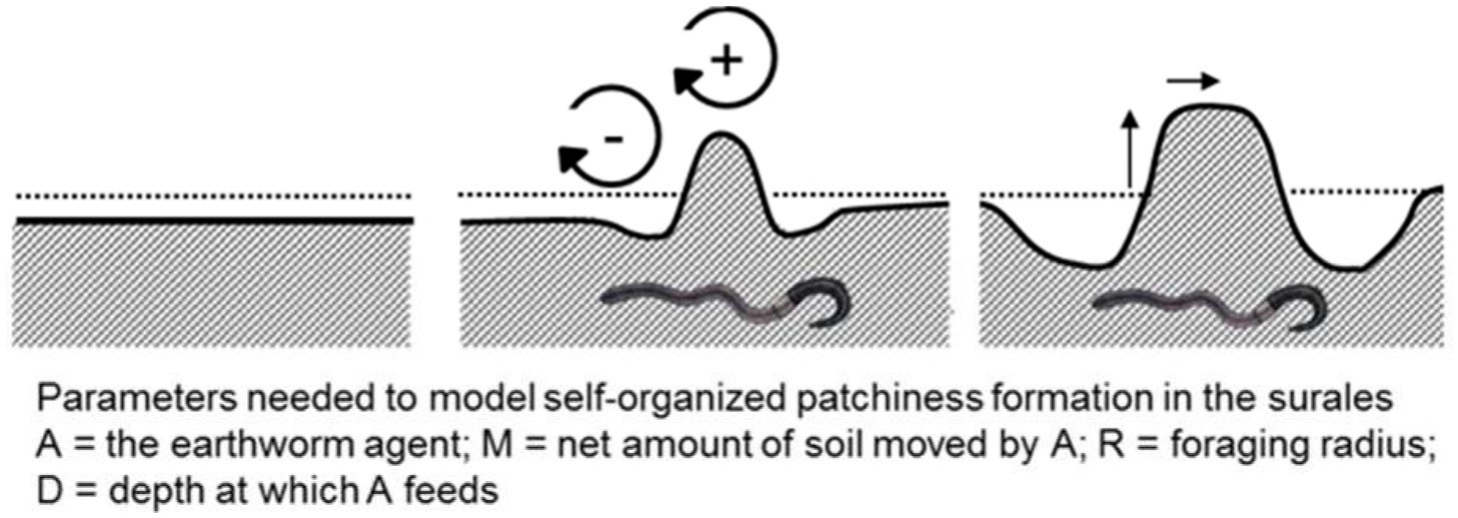
Mechanisms leading to spatial self-organization in *surales* landscapes. Simplistic model showing how self-organized mechanisms can lead to the emergence of *surales* mounds and the parameters needed to implement the model.

Our study provides convincing evidence that the formation of *surales* mounds is explained by the activity of bioturbating earthworms, in our sites *Andiorrhinus* sp. A combination of four features of the behavior of this species can explain how mounds are initiated and grow: (1) This earthworm feeds in flooded soils (and is absent from non-flooded sites). (2) Like some other earthworms in flooded habitats [94–98], individuals excrete large volumes of casts to build towers from which they emerge above the water surface to respire. (3) Individuals of *Andiorrhinus* sp. were exclusively found in large vertical burrows, located directly below their surface casts on mounds (Fig 8). This observation suggests that individuals use a permanent gallery system to return repeatedly to the same tower, where they continue to excrete large volumes of casts (Fig 10). This in turn indicates that individual earthworms have a restricted foraging radius and can be considered (like the temperate-zone species *Lumbricus terrestris* [99]), to be central-place foragers. (4) Individual earthworms continue to use, and add to, the constructions of other individuals. The lifespan of individuals of *Andiorrhinus* sp. is unknown, but is certainly much shorter than the time required for the accumulation of casts such as those shown in Fig 8. Such transgenerational ‘ecological inheritance’ of constructions is known for the best-studied of all earthworms, *Lumbricus terrestris* [100]. Elevated structures should be a highly valuable resource for *Andiorrhinus* sp. in seasonal wetlands, and individual earthworms that preferentially colonize pre-existing mounds should greatly benefit. We posit that these four behavioral traits have the potential to generate the net displacement of ingested soil from the areas immediately surrounding mounds to their top, resulting in the growth of mounds and simultaneously in the lowering of the soil surface level in the area surrounding the mound (Fig 11). These features of the foraging behavior of earthworms thus seem to produce the key mechanism leading to the formation of *surales* landscapes. Much remains to be learned about the ecological strategy of *Andiorrhinus* sp. and its feeding and burrowing behavior. In its use of a vertical burrow system, it resembles anecic species, but its feeding behavior is that of a geophagous species. It is perhaps best classed, like *Polypheretima elongata* [101], as an endoanecic species. More precise information on its foraging radius, rates of soil ingestion and of deposition of casts in soil and on the soil surface are required for quantitative modelling of *surales* formation and spatial self-organization.

The mechanisms that we propose to explain the great spatial regularity of *surales* earth-mound landscapes are analogous to those that lead to spatial self-organization in semi-arid shrublands [2,3]. In the latter landscapes, a key resource, water, strongly limits plant growth. However, plants modify the distribution of this resource. They concentrate it locally where their roots create porous soil in which water infiltrates and vegetation patches develop; and they deplete water between these patches, where water runs off laterally over encrusted soil and there is severe competition for the water that does infiltrate. The combination of a positive effect of plants on water availability (facilitation) at short scale and a negative effect (competition) at a somewhat longer scale leads to formation of regularly spaced patches, in a way first modeled by Turing [102]. In the case of *surales*, the key limiting resource is well-aerated soils during the flooding season. In shallowly flooded soils, *Andiorrhinus* sp. builds elevated structures (towers, which become mounds) to respire, creating patches of this resource. As the mound grows through movement of soil to it from the surrounding area, the basin near the mound gets deeper, decreasing the probability that a new small mound can emerge within a threshold minimal distance of an existing mound, a distance determined by the foraging radius of individual earthworms (Fig 11). In this model, the feedback effect of earthworms on topography is positive at the short scale (creation of the mound) and negative at a slightly longer scale (deepening of the basin around the mound). Thus, as in semi-arid shrublands [2,3], Turing-like mechanisms may underlie the formation of these earth-mound landscapes.

### Contributions of various ecosystem engineers to the formation of larger *surales* mounds

Morphological analysis of soil aggregates indicated that although biostructures in smaller mounds (Site 1) are predominantly produced by the earthworm *Andiorrhinus* sp. (accompanied by small quantities of aggregates produced by social insects), diverse soil engineer organisms—additional earthworm species, but predominantly plant roots—contribute along with *Andiorrhinus* sp. to soil structure in the higher, broader mounds of Sites 2 and 3. For instance, in the largest mounds (Site 3), we showed that roots played a key role in formation of soil structure, with root-associated aggregates accounting on average for 53.2% of the macroaggregates of the soil matrix. These observations support the hypothesis that *Andiorrhinus* sp. initiates *surales* formation, creating favorable well-drained habitat that can then be colonized by other earthworms and by plants. While *Andiorrhinus* sp. dominates earthworm biomass in mounds of all sizes, other engineers may contribute to growth of mounds and, by reducing soil erosion [103], to their maintenance.

### Diversity among *surales* landscapes represents a chronosequence of ecosystem development

**Fig 12:**
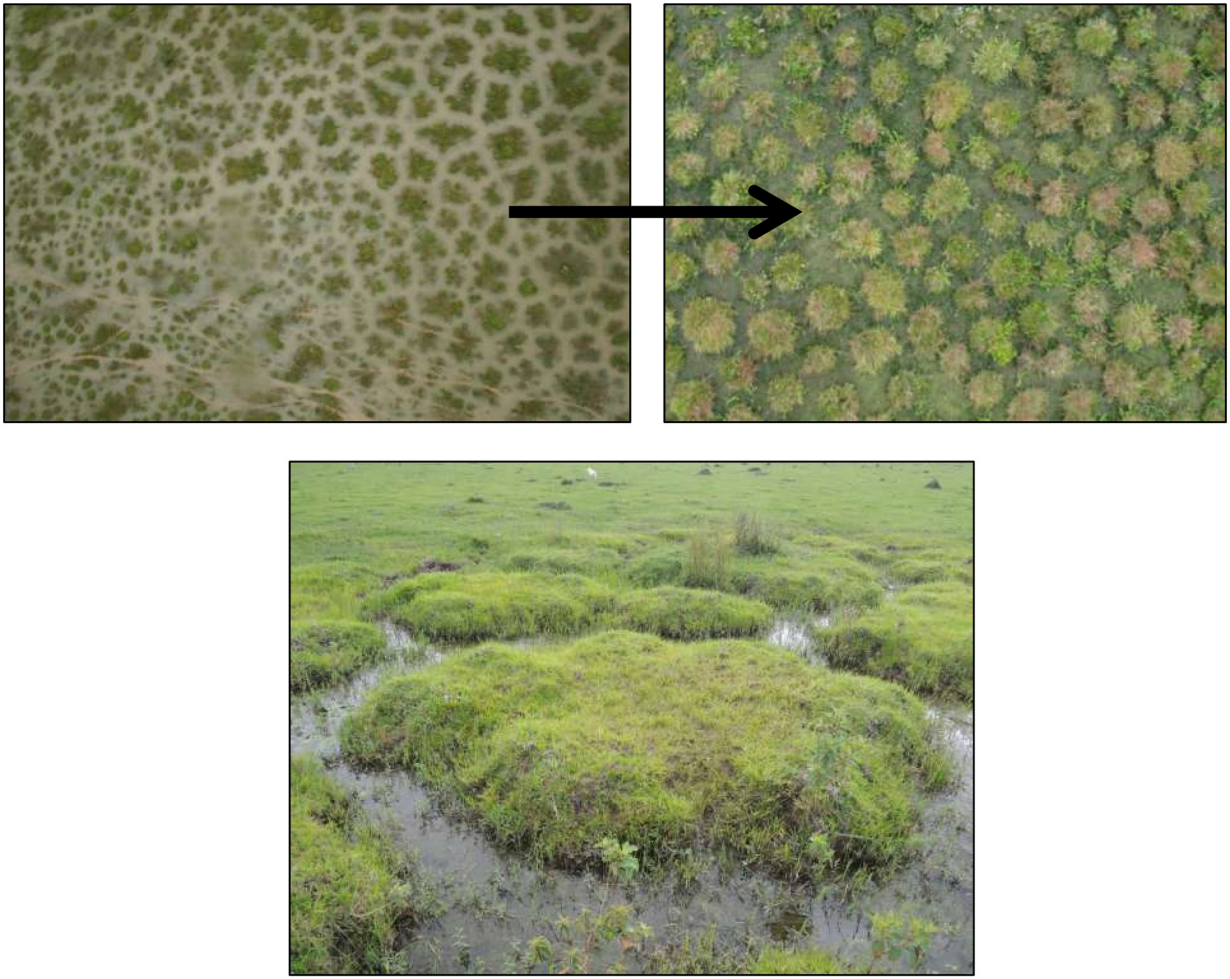
Small *surales* mounds coalescing into larger mounds. Top left. Aerial photograph taken using the Pixy^™^ drone (Delphine Renard 2012) of small mound in coalescing *surales* developing stage in Site 1. Top right. Aerial photograph taken using the Pixy^™^ drone (Delphine Renard 2012) of larger mounds in Site 2. Bottom. Coalescing *surales* mounds forming a larger mound in Site 2. Photo Doyle McKey 2012.

Within the area sampled in each of the three *surales* sites we studied, mounds were all of similar size and shape and featured similar vegetation cover, earthworm communities and macroaggregate composition in the upper levels of the soil matrix. However, among the three sites, and among areas within them, there was great variation in all these parameters. We suggest that our results show this variation among sites to represent a gradient of mound development, marked by increasing aggradation of soils in mounds over time, with corresponding degradation (soil subsidence) in the area surrounding each mound. Five arguments support this hypothesis (1) **Geomorphology**. The smallest mounds are low and flat, and occur in shallowly flooded basins. These coalesce into broader, but still low and flat, mounds, which then grow even broader and taller, with narrower, but deeper, spaces between mounds. (2) **Soil morphology**. As mounds grow broader and taller, they contain an increasingly large proportion of biogenic macroaggregates, indicating the importance of bioturbation and aggradation in mound development. At all stages, mound soils have greater contents of biogenic aggregates than do inter-mound soils. The small, low mounds that first appear are comprised entirely of fresh earthworm casts. In the larger mounds, fresh earthworm casts are concentrated at the periphery of the mound, suggesting that mounds grow as small mounds coalesce and earthworms continue to forage in the adjacent basin, depositing casts on the growing mounds (Fig 12). (3) **Plant communities**. As mounds grow broader and taller, plant species assemblages become more differentiated between mound and inter-mound habitats and the diversity of plant species on mounds increases, until woody species become dominant and a few species of trees comprise most of the vegetation on the largest mounds (Site 3). As the inter-mound basin gets deeper, it is flooded for a longer period each year. Aquatic plants increase in frequency and diversity, then decline where trees on mounds create shady conditions in the basin. (4) **Earthworm communities**. Species composition of earthworm communities varies across stages of growth, and species richness increases in the initial stages. *Andiorrhinus* sp. dominates in contribution to biomass at all stages, and aside from scattered individuals of *Ocnerodrilus occidentalis*, *Andiorrhinus* sp. is the only species found in inter-mound as well as in mound soils. (5) **Soil phytoliths**. Soils of the tree-covered mounds of Site 3, which virtually completely lack herbaceous vegetation today (scattered individuals of a single sedge species), show phytolith composition typical of earlier stages in the hypothesized chronosequence, even in the top few centimeters of the soil profile. Landowners in Site 3 reported that around 1950 the site was covered by herbaceous vegetation, confirming this vegetational change. Succession from dominantly herbaceous vegetation to tree cover was apparently too rapid to allow a detectable accumulation of phytoliths from the recent tree vegetation cover near the surface, particularly given the high rates of bioturbation.

### Microtopographical variation and the initiation and development of *surales* mounds

**Fig 13:**
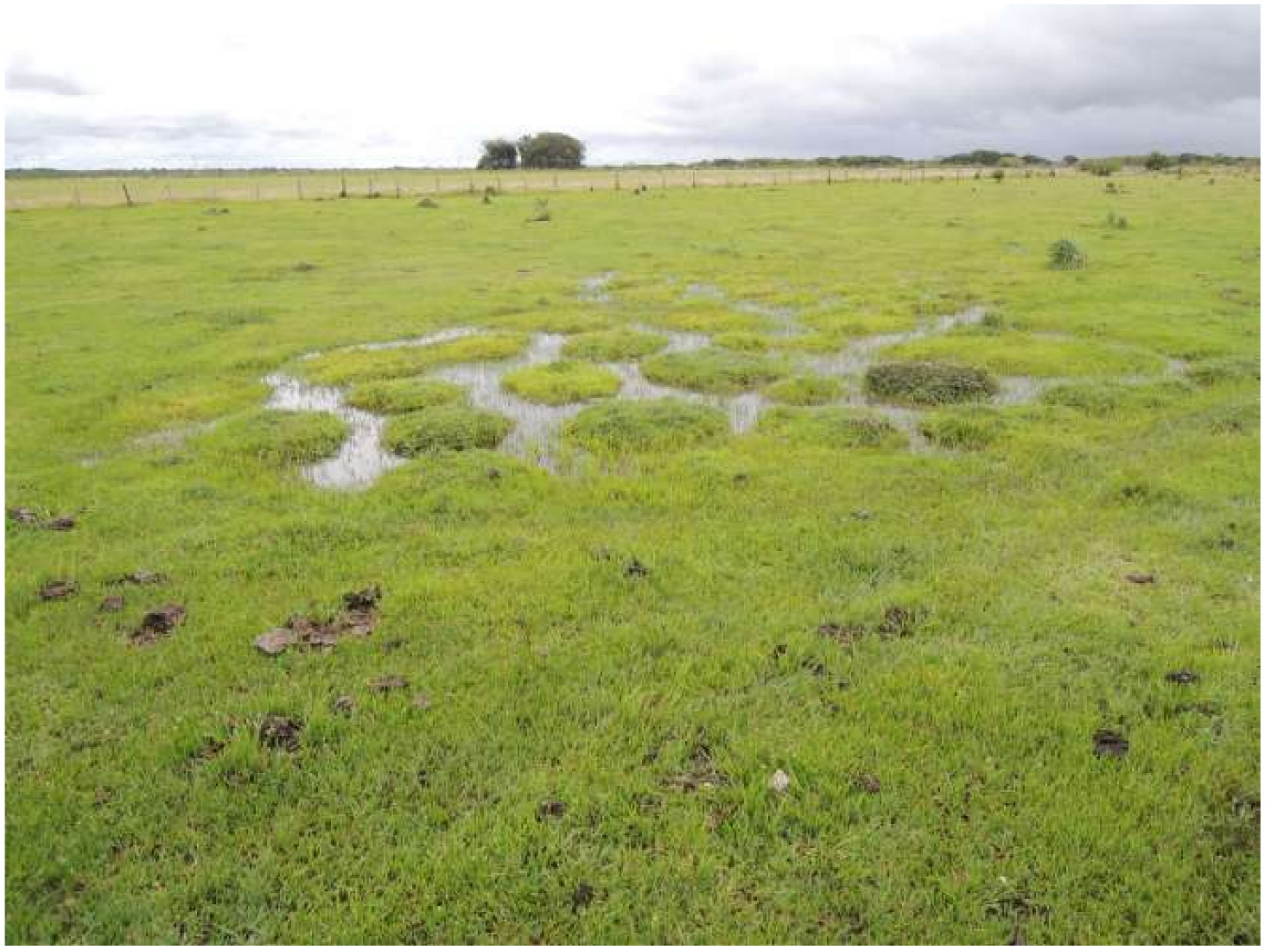
Influence of topographical depressions on *surales* development. Small basin containing *surales* in Site 1. Photo Doyle McKey 2012.

Field data, aerial photographs and satellite imagery all show that *surales* landscapes form only within a narrow range of flooding depths. In the absence of flooding, mounds do not form. *Andiorrhinus* sp. may simply not be present in such sites, and if it is, it would gain no advantage from mound construction. In shallowly flooded areas, *Andiorrhinus* sp. constructs mounds and the *surales*-forming process is initiated. However, when flooding exceeds a certain depth, mounds are either not initiated or do not persist through the high-water season. The threshold depth beyond which mounds do not form is not known with certainty, but the smallest *surales* mounds we observed were in basins that were flooded by less than about 15 cm during the high-water season. *Surales* appear to form readily in such shallowly flooded areas, even in very small depressions in otherwise unflooded areas (Fig 13). The threshold depth is presumably related to the maximum height of the initial tower the earthworm can build, but precisely what mechanism sets the threshold depth for *surales* initiation is unknown. Are towers initiated in still-waterlogged soils during the dry season, and only those persist that reach the minimum height needed to remain above floodwaters through the rainy season? Or are the incipient mounds initiated in the rainy season, being built up in already flooded soil?

**Fig 14:**
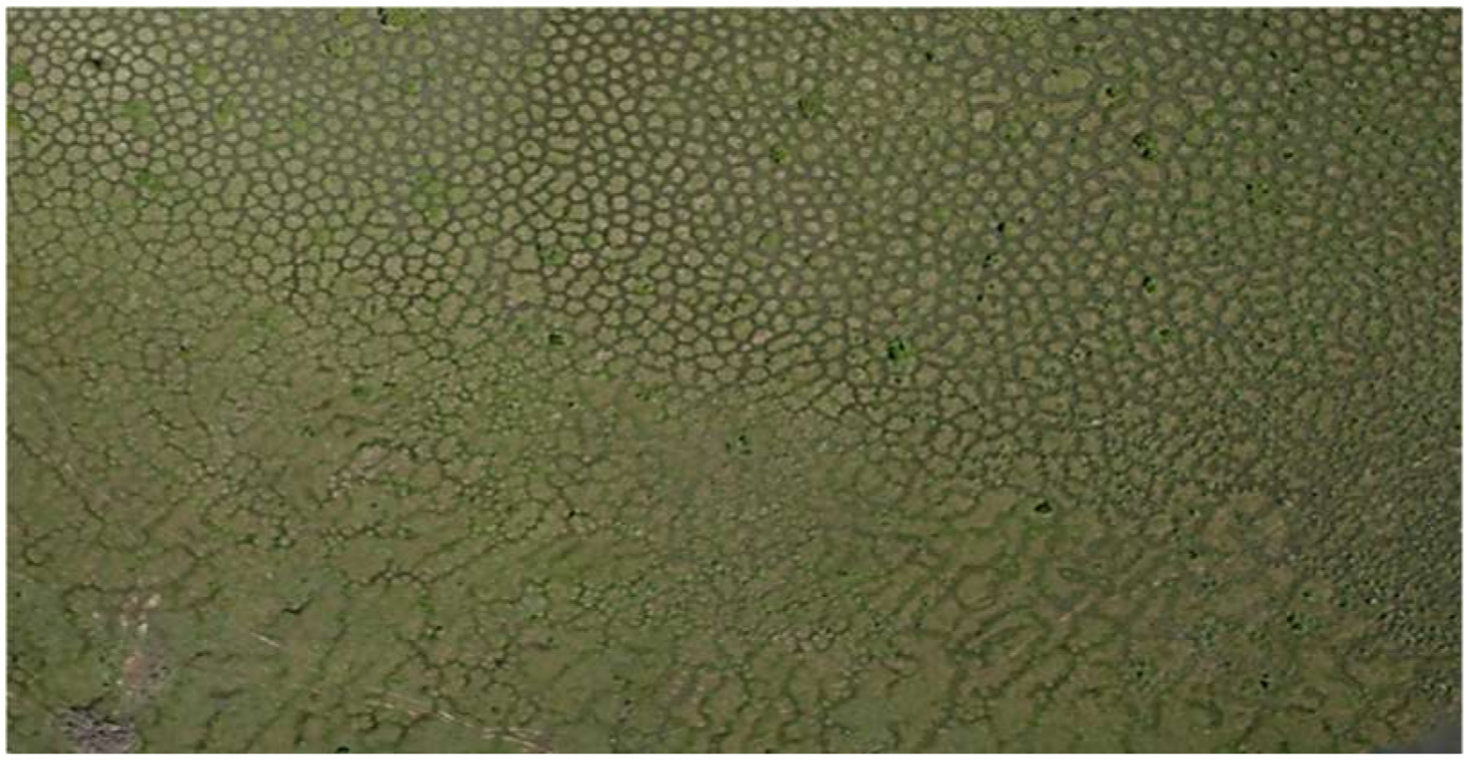
Aerial photograph of Site 2 showing the same sequence of change in surales mound patterning—from spots to labyrinth and gaps, happening at the scale of a hundred meters—predicted by models of self-organization developed for other ecosystems. Photograph taken using Pixy^™^ drone, Delphine Renard 2012.

Within the range of flooding depth in which *surales* form, local topographic variation appears to influence the form taken by *surales* mounds. Within our field sites, particularly Site 2, in addition to round structures, we observed a great diversity of shapes and patterns in *surales* mounds, a diversity that is also visible in many other areas within the Orinoco Llanos (GE visit). Fig 14 shows that *surales* in Site 2 took forms ranging from spotted to labyrinthic, the variation corresponding to a topographical gradient from the center to the margin of the basin. Whereas process-based models can account for the range of forms observed in patterned vegetation in semi-arid shrublands [3], mechanisms leading to labyrinthic patterns in *surales* landscapes have not been investigated. Small differences in elevation over this gradient can affect the period and the depth of flooding, but precisely how this environmental variation affects the strength and spatial scale of feedback effects of earthworms, and thereby the shape of mounds, is a subject for further study. Modelling the bioturbation effected by *Andiorrhinus* sp., integrating more detailed information on the behavior and activity of this earthworm and taking into account micro-topography and eco-hydrological functioning of the seasonal floodplains within which *surales* form, could provide a better understanding of how self-organizing processes generate the vast and complex mosaic of *surales* landscapes in the Orinoco Llanos.

### Emerging questions

If variation in the size and shape of *surales* mounds does reflect, at least in part, different stages of development, why then are all sites that bear *surales* not covered with hypothesized advanced stages, like the large tree-covered mounds of Site 3? The continued existence of putative early stages suggests that there exist processes that generate new, flat, shallowly flooded areas in which *surales* are initiated. What are these processes? Today, disturbances related to changes in land use by humans produce new areas in which *surales* can form. For example, since the creation of an airstrip for small planes ten years ago near an oil-well installation neighboring Site 2, small *surales* mounds have appeared in the drainage ditches parallel to the strip. Also, many areas of *surales* are being graded to produce rice paddies. Abandoned rice paddies could produce large areas where conditions favor initiation of *surales*. At longer time scales, given the restriction of *surales* formation to a narrow range of flooding depths, hydrological changes could reset the process. Shifting fluvial dynamics, for example, could lead to sedimentation of more deeply flooded areas, favoring the initiation of *surales* in areas formerly occupied by *esteros*, and to shallow flooding of areas that previously were not subject to flooding. Cycles of variation in rainfall at scales of decades or centuries could have similar effects.

Other questions arise from the hypothesis that the mosaic observed today partly reflects a sequence of development: whether or not there is an ‘ultimate’ stage of *surales* development, and if so what this ‘equilibrium state’ might resemble, are unknown. For example, *surales* mounds like those in Site 3 may continue to grow, producing mounds even larger than those so far observed. On the other hand, the erosion that produces ‘mushroom-shaped’ mounds in this site (Fig 1) could eventually lead to collapse of mounds. Do *surales* mounds then ‘die’? In the largest mounds we found, including those eroded at the base, *Andiorrhinus* sp. was still present and large amounts of its fresh casts still occurred on mound surfaces. Environmental differences may influence how *surales* develop, so that any putative ‘equilibrium state’ could differ among sites. For example, does the importance of erosion vary among sites, depending on the relative influence of river flow and of local rainfall on flooding dynamics? We observed that *surales* mounds in Site 3 were close to a permanent stream and the presence of fish in the inter-mound basin during the dry season indicated water flow between the stream and the *surales* basin. Does greater erosion by water explain the peculiar shape of mounds in this site? Finally, it is unknown how long it takes for *surales* to develop from small, flat-topped grass-covered mounds to large tree-covered mounds.

Finally, a prediction arising from our results is that large earthworms may have driven the development of similar patterned landscapes in seasonal shallowly flooded or waterlogged wetlands elsewhere in the tropics. The similarity between *surales* landscapes and the *sartenejales* landscapes of Bolivia’s Beni savannas [96] was already noted by Hanagarth [97]. Several intriguing and ecologically virtually unknown earthworm-fashioned landscapes are documented from Uganda [94], South Africa [98] and New Guinea [95]. Comparative study of these landscapes and the worms that make them would be most enlightening.

## Supporting Information

S1 Fig Sampling design for phytoliths in Site 1 and 2. Vertical dotted lines represent the trenches in which we took phytolith samples from profiles.
(TIF)

S1 Table Plant species encountered during the dry season in the point-intersect line transects in Sites 1, 2 and 3, and their contribution to the percentage of cover abundance in *surales* mound and inter-mound habitats. (DOCX)

S2 Table Plant species encountered during the wet season in the point-intersect line transects in Sites 1, 2, 3 and 5, and their contribution to the percentage of cover abundance in *surales* mound and inter-mound habitats. (PDF)

S3 Table Tree species encountered in Site 3 during the dry and the wet season. (DOCX)
