# Supplementary figures and images for: The Surales, Self-Organized Earth-Mound Landscapes Made by Earthworms in a Seasonal Tropical Wetland
Source: PLoS One. 2016 May 11;11(5):e0154269. doi: 10.1371/journal.pone.0154269 (PMC4864223; doi:10.1371/journal.pone.0154269)

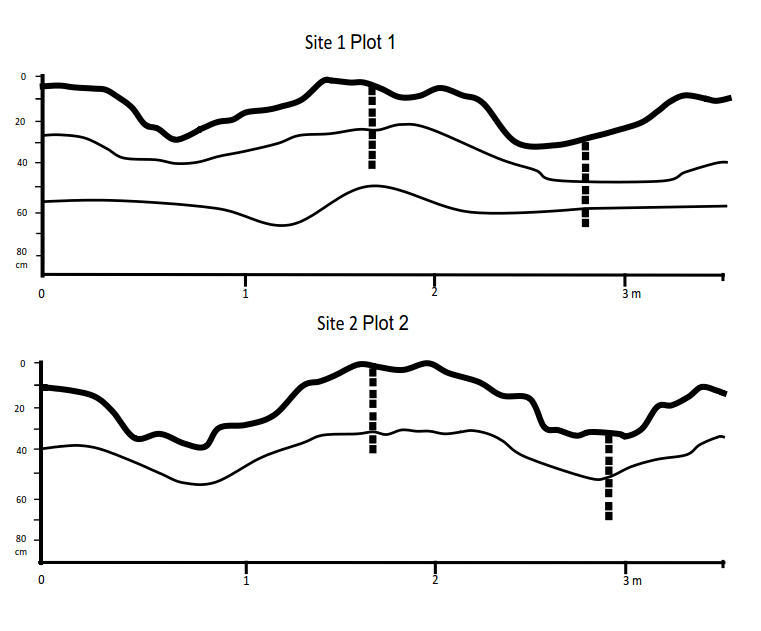

Supplement: S1 Fig — Vertical dotted lines represent the trenches in which we took phytolith samples from profiles. (TIF) [file pone.0154269.s001.tif]
