# Supplementary material for: The Surales, Self-Organized Earth-Mound Landscapes Made by Earthworms in a Seasonal Tropical Wetland
Source: PLoS One. 2016 May 11;11(5):e0154269. doi: 10.1371/journal.pone.0154269 (PMC4864223; doi:10.1371/journal.pone.0154269)
Supplement: S1 Table — (DOCX) [file pone.0154269.s002.docx]

|  | **Cover abundance (%)** | | | | | |
| --- | --- | --- | --- | --- | --- | --- |
|  | **Site 1** | | **Site 2** | | **Site 3** | |
| **Plant Species** | Inter-mounds (129 contacts) | Mounds (480 contacts) | Inter-mounds (156 contacts) | Mounds (526 contacts) | Inter-mounds (18 contacts) | Mounds (82 contacts) |
| *Abildgaardia* cf. *ovata* (Burm. f.) Kral | - | - | 7.69 | 0.38 | - | - |
| *Acanthella* sp. | - | 0.42 | - | 0.38 | - | - |
| *Acanthospermum* sp. | - | 0.42 | - | - | - | - |
| *Acisanthera* cf. *uniflora* (Vahl) Gleason | - | - | 0.64 | 1.90 | - | - |
| *Alysicarpus* sp. | - | - | 1.28 | - | - | - |
| *Andropogon bicornis* L. | 0.78 | 12.92 | - | - | - | - |
| *Aristida capillacea* Lam. | - | 3.33 | 2.56 | 20.53 | - | - |
| *Aristolochia claussenii* Duch. | - | 1.46 | - | - | - | - |
| *Aspidosperma* cf. *parvifolium* A. DC. | - | 0.83 | - | - | - | - |
| *Axonopus purpusii* (Mez) Chase | - | 9.17 | 0.64 | 23.95 | - | - |
| *Becquerelia* sp. | - | 0.42 | - | - | - | - |
| *Bernardia* sp. | - | 0.21 | - | 1.90 | - | - |
| *Centrosema* sp. | 0.78 | - | - | - | - | - |
| *Connarus venezuelanus* Baill. | 0.78 | - | - | - | - | - |
| *Curatella americana* L. | - | 0.42 | - | - | - | - |
| *Cyperus esculentus* L. | 5.43 | 7.71 | - | - | - | - |
| *Cyperus haspan* L. | - | 0.21 | - | - | - | - |
| *Cyperus luzulae* (L.) Rottb. ex Retz. | - | 1.67 | - | - | - | - |
| *Cyperus* sp. | 17.05 | 1.04 | - | - | - | - |
| *Davilla nitida* (Vahl) Kubitzki | 0.78 | 9.17 | - | - | - | - |
| *Declieuxia* cf. *fruticosa* (Willd. ex Roem. & Schult.) Kuntze | 0.78 | 0.63 | 0.64 | 0.38 | - | - |
| *Desmodium adscendens* (Sw.) DC. | 2.33 | 0.42 | - | - | - | - |
| *Desmodium barbatum* (L.) Benth. | - |  | - | - | - | - |
| *Diodia* cf. *apiculata* (Willd.) K. Schum. | - | - | - | 1.14 | - | - |
| *Eichhornia azurea* (Sw.) Kunth | - | - | 1.28 | - | - | - |
| *Eleocharis* sp. | - | - | 1.28 | - | - | - |
| *Elephantopus mollis* Kunth | - | 0.83 | - | 1.71 | - | - |
| *Eriocaulon* sp. | - | - | 1.28 | 12.36 | - | - |
| *Fimbristylis* cf. *complanata* (Retz.) Link | - | - | 1.92 | 2.66 | - | - |
| *Fimbristylis* sp. | - | - | - | 0.19 | - | - |
| *Hydrolea spinosa* L. | - | - | 5.77 | 0.38 | - | - |
| *Hyptis brachiata* Briq. | - | 1.04 | - | - | - | - |
| *Hyptis* sp. | - | - | 8.33 | - | - | - |
| *Hyptis* sp. 2 | 3.88 | 2.50 | - | - | - | - |
| *Hyptis* sp. 3 | 3.10 | 0.42 | - | - | - | - |
| Undetermined *1* | - | - | 3.21 | - | - | - |
| Undetermined 2 | - | - | - | 0.76 | - | - |
| Undetermined 3 | - | - | 0.64 | - | - | - |
| *Kyllingia* sp. | - | - | - | - | - | - |
| *Leandra* cf. *aristigera* (Naudin) Cogn. | - | 0.63 | - | - | - | - |
| *Leersia hexandra* Sw. | 15.50 | 1.46 | 0.64 | - | - | - |
| *Limnosipanea* sp. 1 | - | - | - | 0.19 | - | - |
| *Limnosipanea* sp. 2 | - | - | - | 0.38 | - | - |
| *Ludwigia erecta* (L.) H. Hara | 2.33 | 0.63 | 1.28 | - | - | - |
| *Lycopodiella* cf. *cernua*(L.) Pic. Serm. | 1.55 | 8.33 | 6.41 | 4.37 | - | - |
| *Mecardonia procumbens* (Mill.) Small | 0.78 | - | - | - | - | - |
| *Melochia villosa* (Mill.) Fawc. & Rendle | - | 2.71 | - | 0.38 | - | - |
| *Miconia albicans* (Sw.) Steud. | - | 1.67 | - | - | - | - |
| *Mimosa pudica* L. | - | 0.42 | - | - | - | - |
| *Mimosa* sp. | - | 1.25 | - | - | - | - |
| Morphospecies 1 | - | - | 7.05 | 0.57 | - | - |
| Morphospecies 2 | - | - | - | 0.95 | - | - |
| Morphospecies 3 | - | - | 0.64 | - | - | - |
| Morphospecies 4 | - | - | 0.64 | 0.76 | - | - |
| Morphospecies 5 | - | - | 0.64 | - | - | - |
| Morphospecies 6 | 23.26 | 8.33 | - | - | - | - |
| Morphospecies 7 | - | 0.42 | - | - | - | - |
| Morphospecies 8 | - | 0.42 | - | - | - | - |
| *Mucuna* sp. | 3.10 | 1.46 | - | - | - | - |
| *Oldenlandia lancifolia* (Schumach.) DC. | 4.65 | 0.83 | 0.64 | - | - | - |
| *Oldenlandia* sp. 1 | - | - | 1.92 | 0.76 | - | - |
| *Oldenlandia* sp. 2 | - | - | - | 0.19 | - | - |
| *Paepalanthus* cf. *lamarckii* | - | - | 4.49 | 1.90 | - | - |
| *Panicum* sp.1 | 6.98 | 0.21 | 2.56 | 2.09 | - | - |
| *Panicum* sp.2 | - | - | 3.21 | 0.38 | - | - |
| *Paspalum* sp. | - | - | - | 0.19 | - | - |
| *Peltaea speciosa* (Kunth) Standl. | 1.55 | - | - | - | - | - |
| *Pennisetum* sp. | - | 0.42 | 12.82 | 3.04 | - | - |
| *Phyllanthus* sp. | - | 0.21 | 0.64 | - | - | - |
| *Pterogastra divaricata* (Bonpl.) Naudin | - | - | - | 3.42 | - | - |
| *Pterolepis* cf. *trichotoma* (Rottb.) Cogn. | - | - | - | 1.90 | - | - |
| *Randia aculeata* L. | - | 1.46 | - | - | - | - |
| *Rhynchospora* cf. *tenuis* Willd. ex Link | - | - | 8.97 | 0.95 | - | - |
| *Rhynchospora nervosa* (Vahl) Boeckeler | - | 1.25 | - | - | - | - |
| *Rhynchospora* sp. | - | - | 2.56 | 1.14 | - | - |
| *Rotala* cf. *ramosior* (L.) Koehne | - | - | - | 0.19 | - | - |
| *Rynchospora cephalotes* (L.) Vahl | - | - | - | - | - | 100.00 |
| *Sabicea* sp. | 0.78 | 5.83 | - | - | - | - |
| *Schizachyrium* sp. | 2.33 | 4.79 | 5.77 | 6.27 | - | - |
| *Sida* sp. | - | - | 1.28 | 0.57 | - | - |
| *Sipanea* sp. | - | - | - | 0.19 | - | - |
| *Tibouchina* cf. *aspera* Aubl. | 0.78 | 1.04 | - | - | - | - |
| *Vigna linearis* (Kunth) Marechal & al. | 0.78 | 0.21 | 0.64 | 0.57 | - | - |
| *Zanthoxylum* cf. *pterota* (L.) Kunth | - | 0.83 | - | - | - | - |

Table S1. Plant species encountered during the dry season in the point-intersect line transects in Sites 1, 2 and 3, and their contribution to the percentage of cover abundance in *surales* mound and inter-mound habitats.
