# Supplementary material for: The Surales, Self-Organized Earth-Mound Landscapes Made by Earthworms in a Seasonal Tropical Wetland
Source: PLoS One. 2016 May 11;11(5):e0154269. doi: 10.1371/journal.pone.0154269 (PMC4864223; doi:10.1371/journal.pone.0154269)
Supplement: S2 Table — (PDF) [file pone.0154269.s003.pdf]

| | Cover abundance (%) | | | | | | |
|---|---|---|---|---|---|---|---|
| | Site 1 | | Site 2 | | Site 3 | | Site 5 |
| **Plant Species** | Inter-mounds (270 contacts) | Mounds (236 contacts) | Inter-mounds (208 contacts) | Mounds (319 contacts) | Inter-mounds (38 contacts) | Mounds (78 contacts) | (302 contacts) |
| *Acisanthera quadrata* Pers. | - | 1.69 | 0.96 | 2.82 | - | - | - |
| *Aeschynomene americana* L. | - | 13.14 | - | 0.94 | - | - | - |
| *Andropogon bicornis* L. | 2.59 | 0.85 | - | 0.31 | - | - | - |
| *Andropogon selloanus* (Hack.) Hack. | - | - | - | 0.31 | - | - | - |
| *Anthurium* sp. | - | 0.42 | - | - | - | - | - |
| *Aristolochia claussenii* Duch. | - | 0.85 | - | 0.31 | - | - | - |
| *Axonopus purpusii* (Mez) Chase | 1.85 | 11.02 | - | 23.20 | - | - | 1.99 |
| *Axonopus* sp. | - | 10.59 | - | 11.29 | - | - | 5.96 |
| *Bernardia* sp. | 2.22 | - | 0.48 | 0.31 | - | - | - |
| *Borreria* sp. | 0.74 | - | 0.96 | - | - | - | - |
| *Calopogonium* cf. *mucunoides* Desv. | 0.37 | 1.69 | - | 3.76 | - | - | - |
| *Clitoria guianensis* (Aubl.) Benth. | - | - | - | 0.31 | - | - | - |
| *Commelina* sp. | - | - | 0.48 | 2.82 | - | - | - |
| *Copaifera pubiflora* Benth. | - | - | - | - | - | 1.28 | - |
| *Curatella americana* L. | - | 1.27 | - | - | - | - | - |
| *Cyperus* sp. | - | - | 0.48 | - | - | - | - |
| *Cyperus* sp. 1 | - | - | 0.48 | - | - | - | - |
| *Cyperus* sp. 2 | - | - | - | 0.31 | - | - | - |
| *Davilla nitida* (Vahl) Kubitzki | - | 1.27 | - | - | - | 5.13 | - |
| *Desmodium barbatum* (L.) Benth. | - | - | - | 0.31 | - | - | 6.62 |
| *Eleocharis* cf. *interstincta* (Vahl) Roem. & Schult. | 1.48 | - | - | - | - | - | - |
| *Eleocharis* cf. *retroflexa* (Poir.) Urb. | - | 1.27 | 6.25 | 0.31 | - | - | - |
| *Elephantopus mollis* Kunth | - | 0.42 | - | 2.51 | - | - | 0.66 |
| *Eriochloa* sp. | - | - | - | 0.31 | - | - | - |
| *Eugenia* sp. | 8.52 | 7.20 | 2.40 | - | - | - | - |
| *Fimbristyles* sp. | - | - | - | - | - | - | 2.98 |

| | | | | | | | |
|---|---|---|---|---|---|---|---|
| *Hydrolea spinosa* L. | 0.37 | - | 2.40 | 0.31 | - | - | - |
| *Hyptis* sp. | - | - | 6.25 | 0.94 | - | - | - |
| *Hyptis* sp. 2 | 4.44 | 0.85 | 10.58 | 0.63 | - | - | - |
| *Hyptis* sp. 3 | 0.74 | - | - | - | - | - | - |
| *Kyllingia* sp. | - | - | - | - | - | - | - |
| *Lantana* sp. | - | 0.85 | - | - | - | - | - |
| *Leersia hexandra* Sw. | 41.48 | - | 25.00 | 2.19 | - | - | 0.33 |
| *Limnocharis* sp. | - | - | 0.48 | - | - | - | - |
| *Lindernia diffusa* (L.) Wettst. | - | - | 0.48 | 0.63 | - | - | - |
| *Ludwigia peploides* (Kunth) P.H. Raven | 1.11 | - | 1.92 | 0.31 | - | - | - |
| *Ludwigia* sp. 1 | 0.37 | - | - | - | - | - | - |
| *Ludwigia* sp. 2 | 0.37 | - | - | - | - | - | - |
| *Lycopodiella* cf. *cernua* (L.) Pic. Serm. | 0.74 | 13.98 | 7.21 | 15.67 | - | - | - |
| *Mabea occidentalis* Benth. | - | - | - | - | - | 3.85 | - |
| *Marsilea minuta* L. | - | - | 0.48 | - | - | - | - |
| *Mayaca* sp. | - | - | 1.92 | - | - | - | - |
| *Melochia* sp. | - | - | 0.96 | 0.31 | - | - | - |
| *Melochia villosa* (Mill.) Fawc. & Rendle | 2.22 | 2.97 | 0.48 | 0.94 | - | - | - |
| *Miconia* sp. 1 | - | - | - | 0.31 | - | 5.13 | - |
| *Mimosa pudica* L. | 0.37 | 7.63 | 1.92 | 1.25 | - | - | 0.33 |
| Morphospecies 1 | - | 0.42 | - | 2.82 | - | - | - |
| Morphospecies 2 | - | - | - | 0.63 | - | - | - |
| Morphospecies 3 | 1.85 | 6.36 | 5.29 | 0.94 | - | - | - |
| Morphospecies 4 | - | - | 1.92 | 2.82 | - | - | - |
| Morphospecies 5 | - | - | - | - | - | - | 1.32 |
| Morphospecies 6 | 4.81 | 2.97 | - | - | - | - | - |
| Morphospecies 7 | - | 0.42 | - | - | - | - | - |
| Morphospecies 8 | - | 0.42 | - | - | - | - | - |
| *Nymphaea* cf. *blanda* Planch. | 10.00 | - | 5.77 | - | - | - | - |
| *Nymphoides indica* (L.) Kuntze | - | - | - | - | 34.21 | - | - |
| *Oldenlandia* sp. | - | - | 1.44 | - | - | - | - |
| *Orthopappus* sp. | - | 0.42 | - | 1.25 | - | - | - |
| *Paspalum* sp. | - | - | - | - | - | - | 66.23 |
| *Paspalum* sp. 1 | - | - | - | 0.63 | - | - | - |
| *Paspalum* sp. 2 | - | - | - | 0.63 | - | - | - |
| *Phyllanthus stipulatus* (Raf.) G.L. Webster | 1.48 | 1.27 | 3.37 | 0.94 | - | - | - |

| | | | | | | | |
|---|---|---|---|---|---|---|---|
| *Pontederia rotundifolia* L. f. | 4.07 | - | 2.40 | - | - | - | - |
| *Psidium* cf. *guianense* Pers. | - | - | - | 6.27 | - | - | - |
| *Rhynchanthera grandiflora* (Aubl.) DC. | - | - | 0.48 | 2.51 | - | - | - |
| *Rhynchospora nervosa* (Vahl) Boeckeler | - | - | - | - | - | - | 13.58 |
| *Rynchospora cephalotes* (L.) Vahl | - | - | - | - | - | 84.61 | - |
| *Sabicea* cf. *villosa* Schult. | - | 7.20 | - | 1.57 | - | - | - |
| *Sagittaria* sp. | - | - | 1.44 | - | - | - | - |
| *Sipanea pratensis* Aubl. | - | 0.42 | 0.48 | 3.13 | - | - | - |
| *Sipanea* sp. | 0.74 | - | 1.44 | 1.25 | - | - | - |
| *Tonina fluviatilis* Aubl. | - | 0.42 | 0.96 | 0.94 | - | - | - |
| *Utricularia radiata* Small | - | - | - | - | 65.79 | - | - |
| *Utricularia* sp. | - | - | 2.40 | - | - | - | - |
| *Utricularia* sp. 1 | 3.70 | - | - | - | - | - | - |
| *Utricularia* sp. 2 | 3.33 | - | - | - | - | - | - |
| *Vernonia* sp. | - | 0.85 | - | - | - | - | - |
| *Zanthoxylum* sp. | - | 0.85 | - | - | - | - | - |

Table S2. Plant species encountered during the wet season in the point-intersect line transects in Sites 1, 2, 3 and 5, and their contribution to the percentage of cover abundance in *surales* mound and inter-mound habitats.
