# Supplementary material for: The Surales, Self-Organized Earth-Mound Landscapes Made by Earthworms in a Seasonal Tropical Wetland
Source: PLoS One. 2016 May 11;11(5):e0154269. doi: 10.1371/journal.pone.0154269 (PMC4864223; doi:10.1371/journal.pone.0154269)
Supplement: S3 Table — (DOCX) [file pone.0154269.s004.docx]

| **DRY SEASON** | **WET SEASON** |
| --- | --- |
| *Clusia* sp. | *Copaifera pubiflora* |
| *Copaifera pubiflora* Benth. | *Davila nitida* |
| *Erythroxylum squamatum* Sw. | *Erythroxylum macrophyllum* |
| *Jacaranda obtusifolia* Bonpl. | *Jacaranda obtusifolia* |
| *Mabea occidentalis* Benth. | *Mabea occidentalis* |
| *Miconia* sp. | *Miconia* sp. |
| Morphospecies 9 | *Siparuna guianensis* Aubl. |
| *Siparuna guianensis* Aubl. |  |

Table S3. Tree species encountered in Site 3 during the dry and the wet season.
